# Supplementary material for: Targeting Triple Negative Breast Cancer Stem Cells by Heat Shock Protein 70 Inhibitors
Source: Cancers (Basel). 2022 Oct 7;14(19):4898. doi: 10.3390/cancers14194898 (PMC9562869; doi:10.3390/cancers14194898)
Supplement: Supplementary file 1 [file cancers-14-04898-s001.zip › File S1.pdf]

Original WB

Targeting Triple Negative Breast Cancer Stem Cells by Heat Shock Protein 70 Inhibitors

Fig. 1C

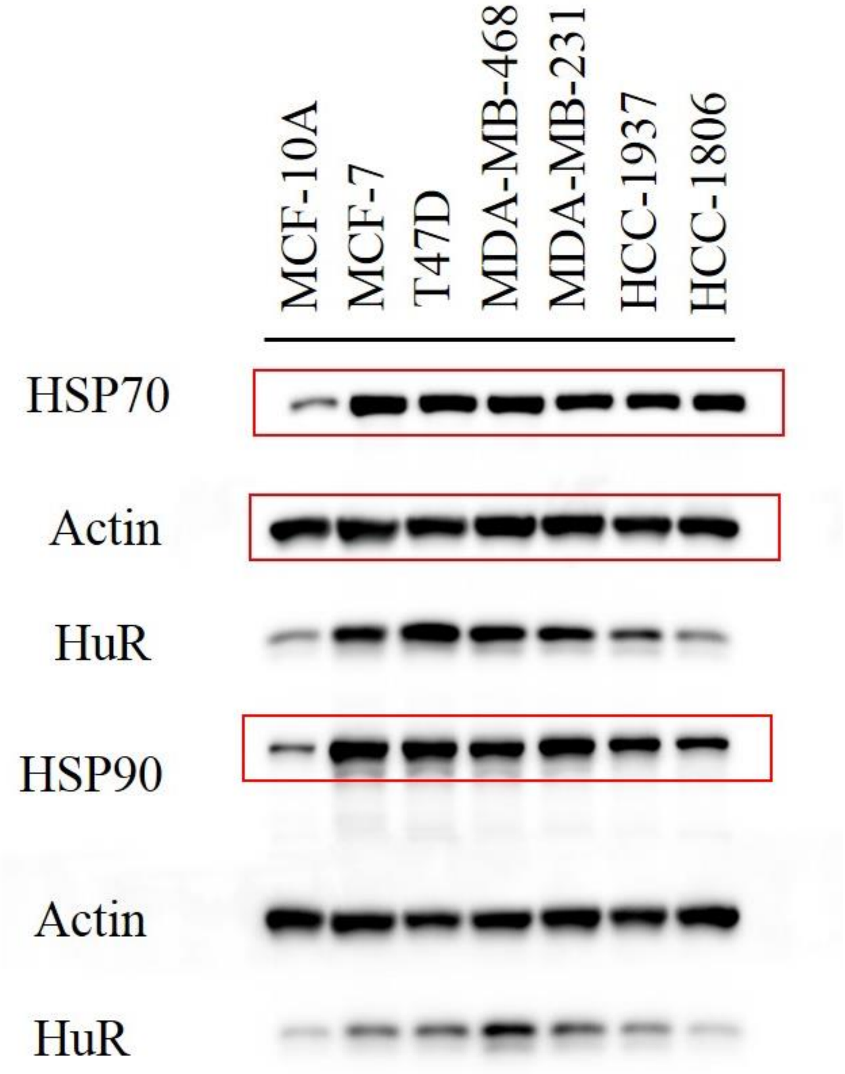
MCF-10A
MCF-7
T47D
MDA-MB-468
MDA-MB-231
HCC-1937
HCC-1806
HSP70
Actin
HuR
HSP90
Actin
HuR

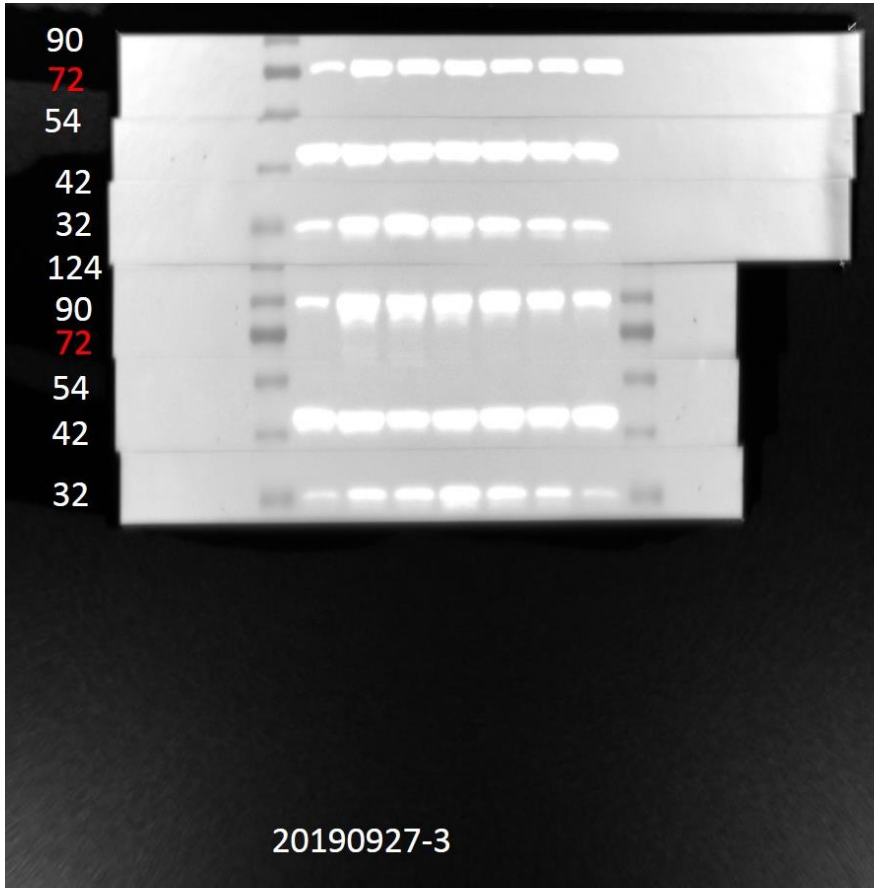
90
72
54
42
32
124
90
72
54
42
32
20190927-3

Fig. 3C

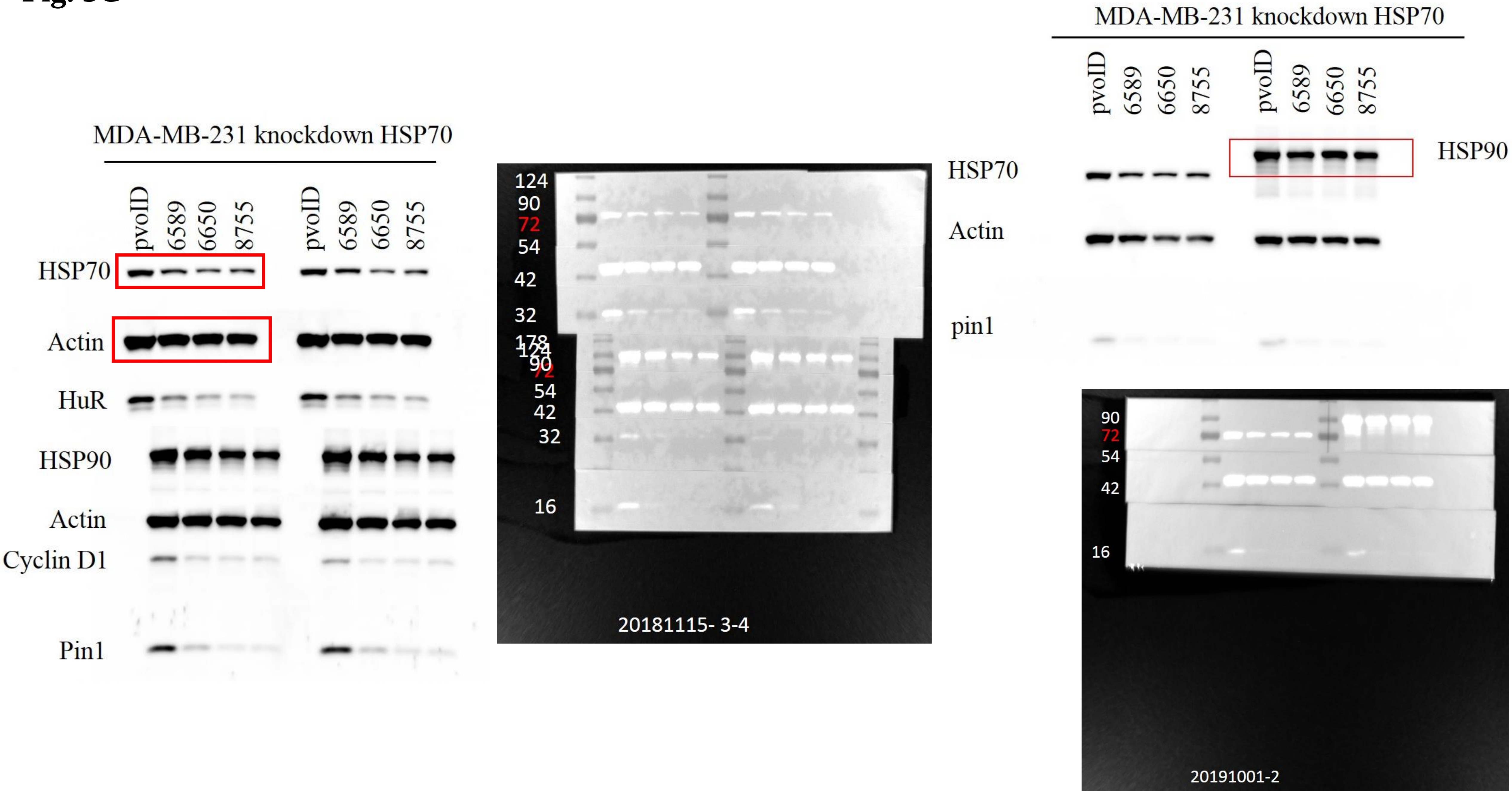
MDA-MB-231 knockdown HSP70
pvoID
6589
6650
8755
HSP70
Actin
HuR
HSP90
Cyclin D1
Pin1
124
90
72
54
42
32
16
20181115- 3-4
pin1
20191001-2

Fig. 4E (left)

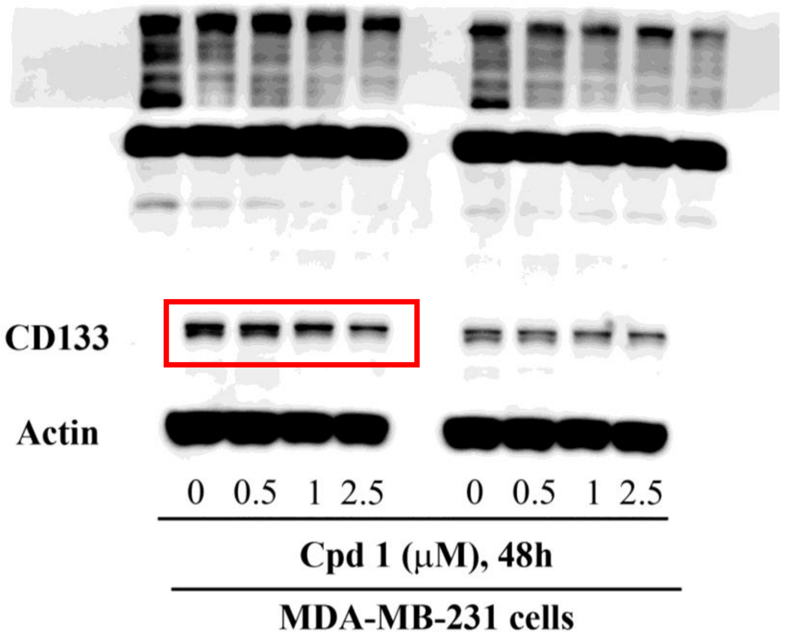
CD133
Actin
0 0.5 1 2.5 0 0.5 1 2.5
Cpd 1 (μM), 48h
MDA-MB-231 cells

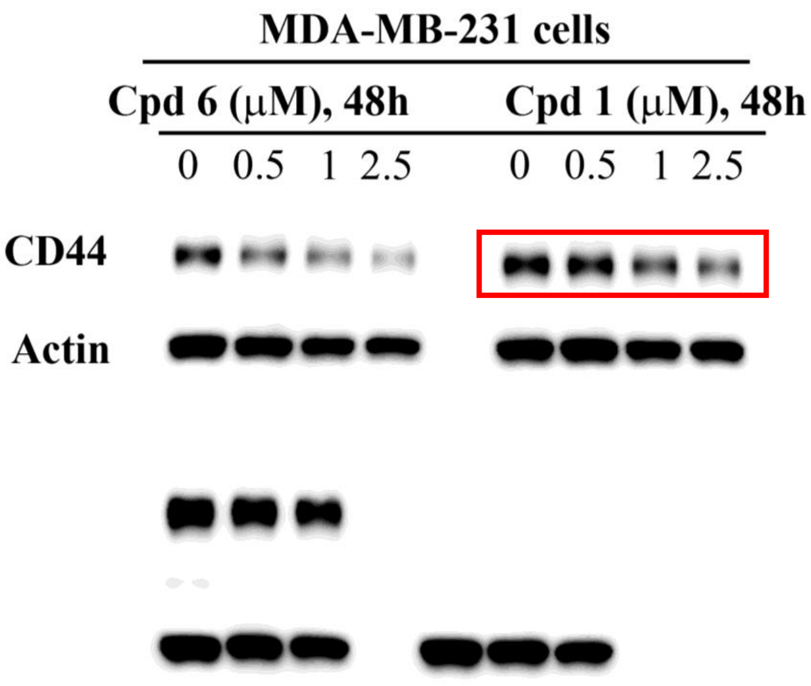
MDA-MB-231 cells
Cpd 6 (μM), 48h
Cpd 1 (μM), 48h
0 0.5 1 2.5 0 0.5 1 2.5
CD44
Actin

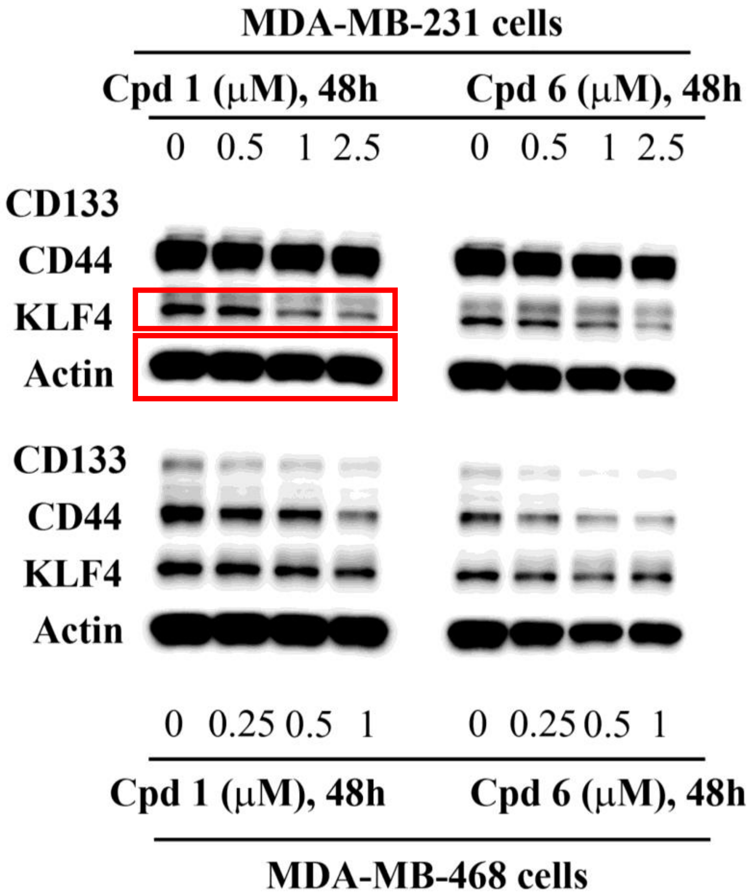
MDA-MB-231 cells
Cpd 1 (μM), 48h
Cpd 6 (μM), 48h
0 0.5 1 2.5 0 0.5 1 2.5
CD133
CD44
KLF4
Actin
CD133
CD44
KLF4
Actin
0 0.25 0.5 1 0 0.25 0.5 1
Cpd 1 (μM), 48h
Cpd 6 (μM), 48h
MDA-MB-468 cells

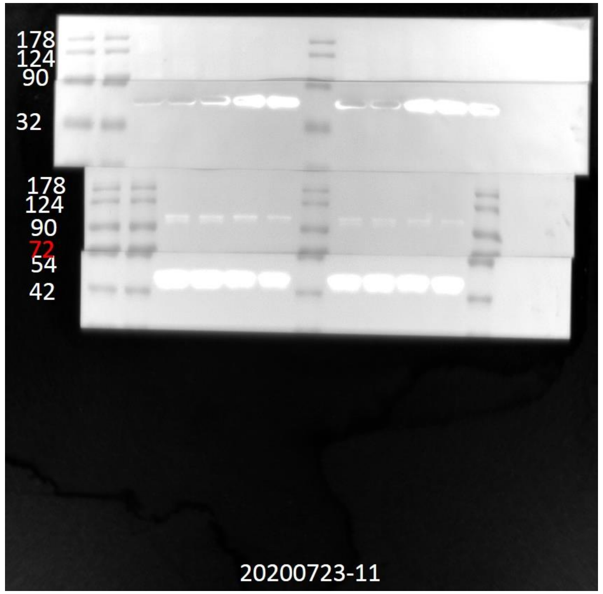
178
124
90
32
178
124
90
72
54
42
20200723-11

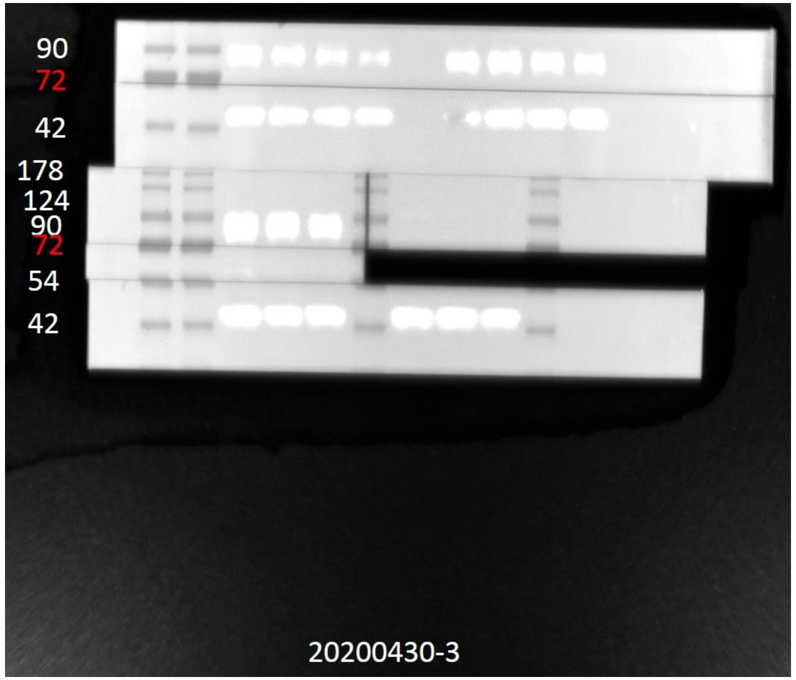
90
72
42
178
124
90
72
54
42
20200430-3

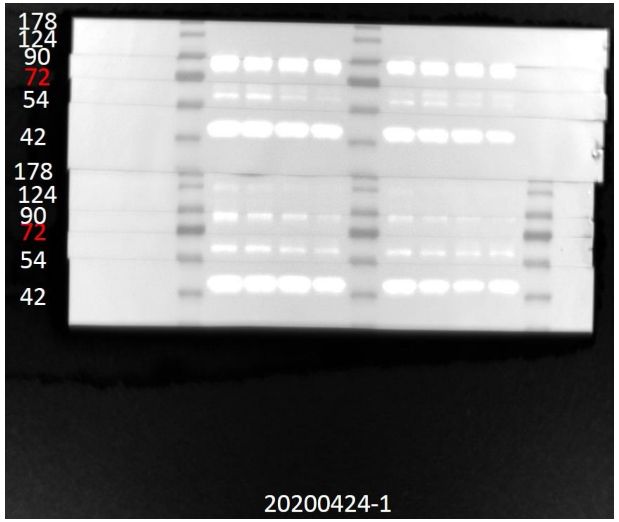
178
124
90
72
54
42
178
124
90
72
54
42
20200424-1

Fig. 4E (left)

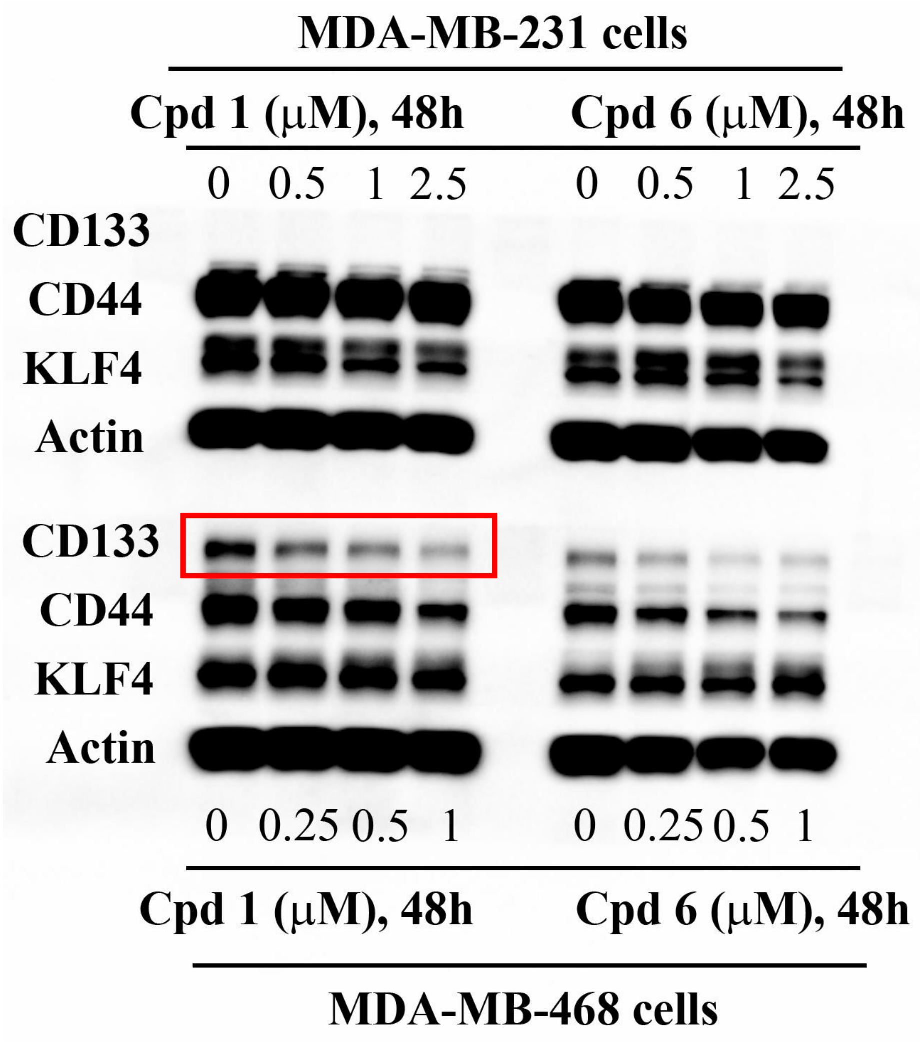
MDA-MB-231 cells
Cpd 1 (μM), 48h
Cpd 6 (μM), 48h
0 0.5 1 2.5
0 0.5 1 2.5
CD133
CD44
KLF4
Actin
CD133
CD44
KLF4
Actin
0 0.25 0.5 1
0 0.25 0.5 1
Cpd 1 (μM), 48h
Cpd 6 (μM), 48h
MDA-MB-468 cells

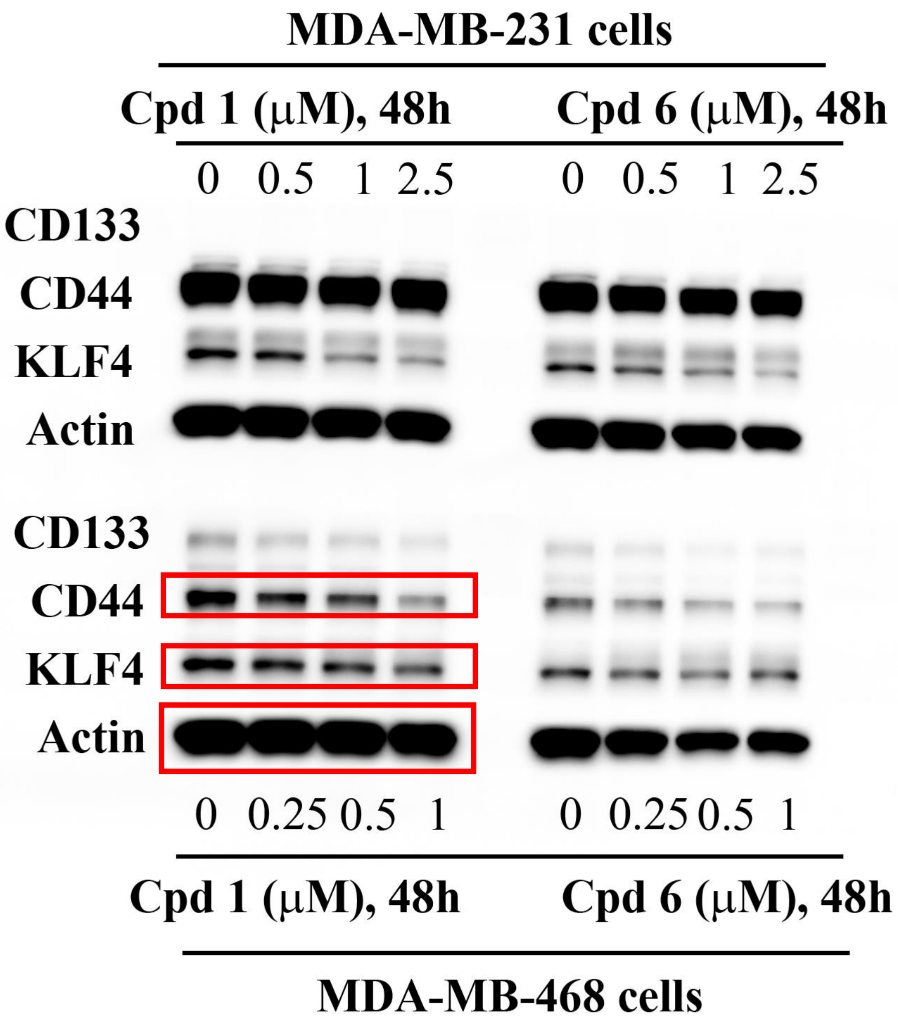
MDA-MB-231 cells
Cpd 1 (μM), 48h
Cpd 6 (μM), 48h
0 0.5 1 2.5
0 0.5 1 2.5
CD133
CD44
KLF4
Actin
CD133
CD44
KLF4
Actin
0 0.25 0.5 1
0 0.25 0.5 1
Cpd 1 (μM), 48h
Cpd 6 (μM), 48h
MDA-MB-468 cells

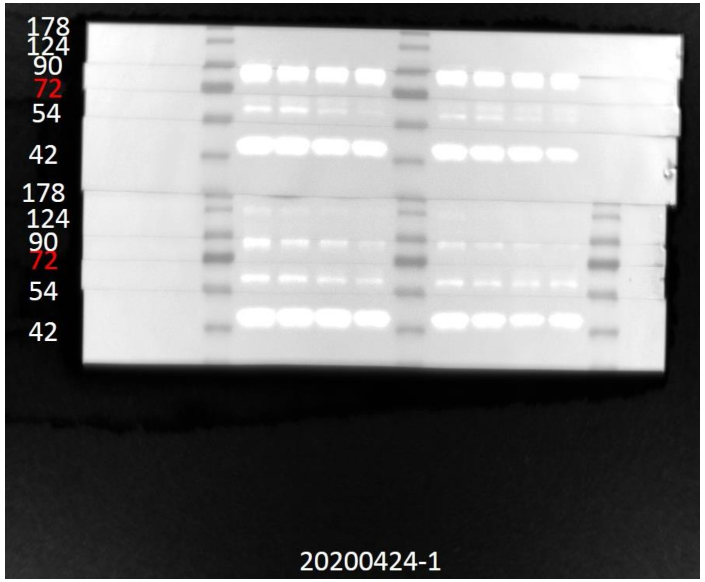
178
124
90
72
54
42
178
124
90
72
54
42
20200424-1

Fig. 4E (right)

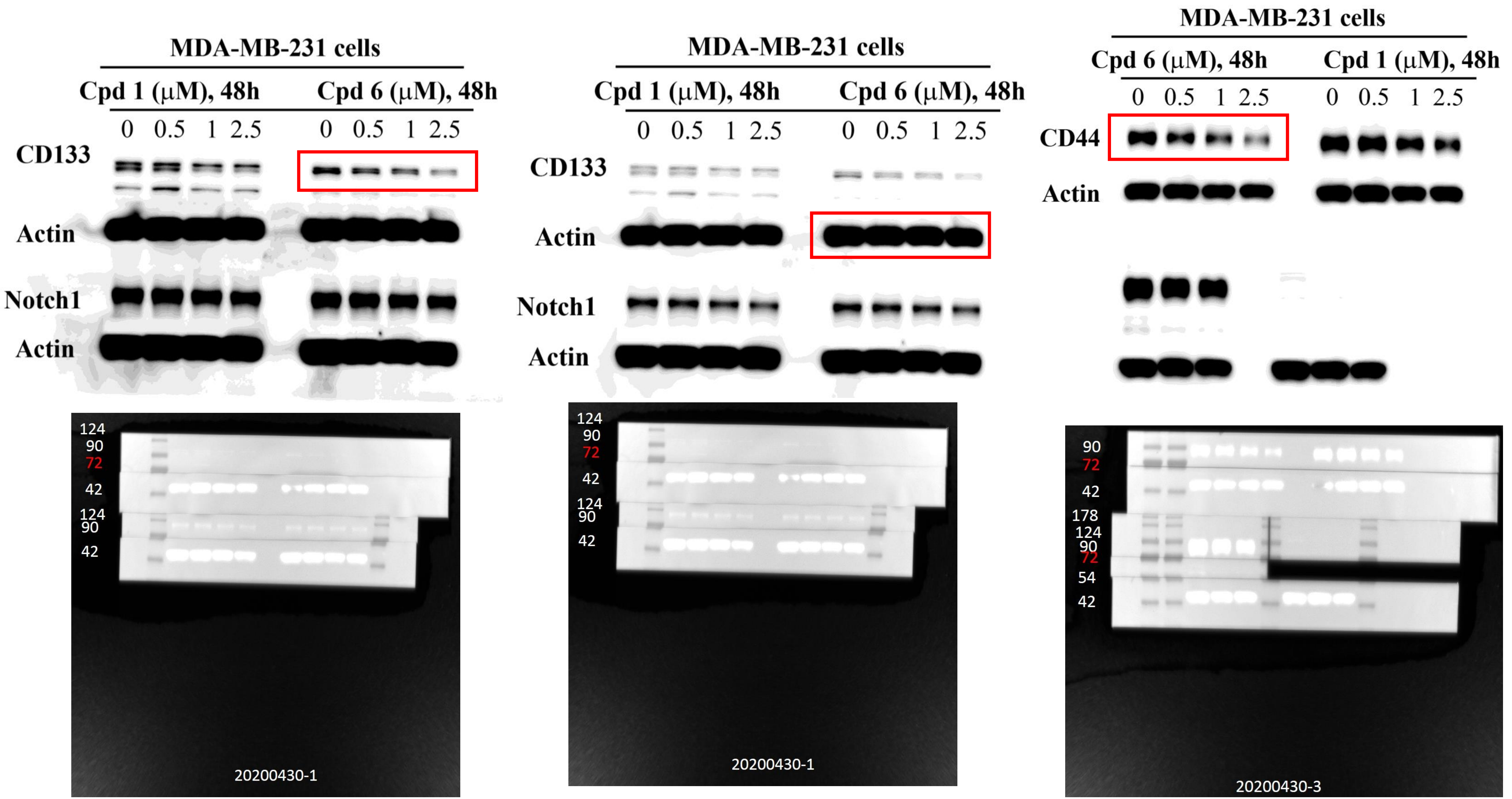
MDA-MB-231 cells
Cpd 1 (μM), 48h
Cpd 6 (μM), 48h
0 0.5 1 2.5
0 0.5 1 2.5
CD133
Actin
Notch1
Actin
MDA-MB-231 cells
Cpd 1 (μM), 48h
Cpd 6 (μM), 48h
0 0.5 1 2.5
0 0.5 1 2.5
CD133
Actin
Notch1
Actin
MDA-MB-231 cells
Cpd 6 (μM), 48h
Cpd 1 (μM), 48h
0 0.5 1 2.5
0 0.5 1 2.5
CD44
Actin
124
90
72
42
124
90
42
20200430-1
124
90
72
42
124
90
42
20200430-1
90
72
42
178
124
90
72
54
42
20200430-3

Fig. 4E (right)

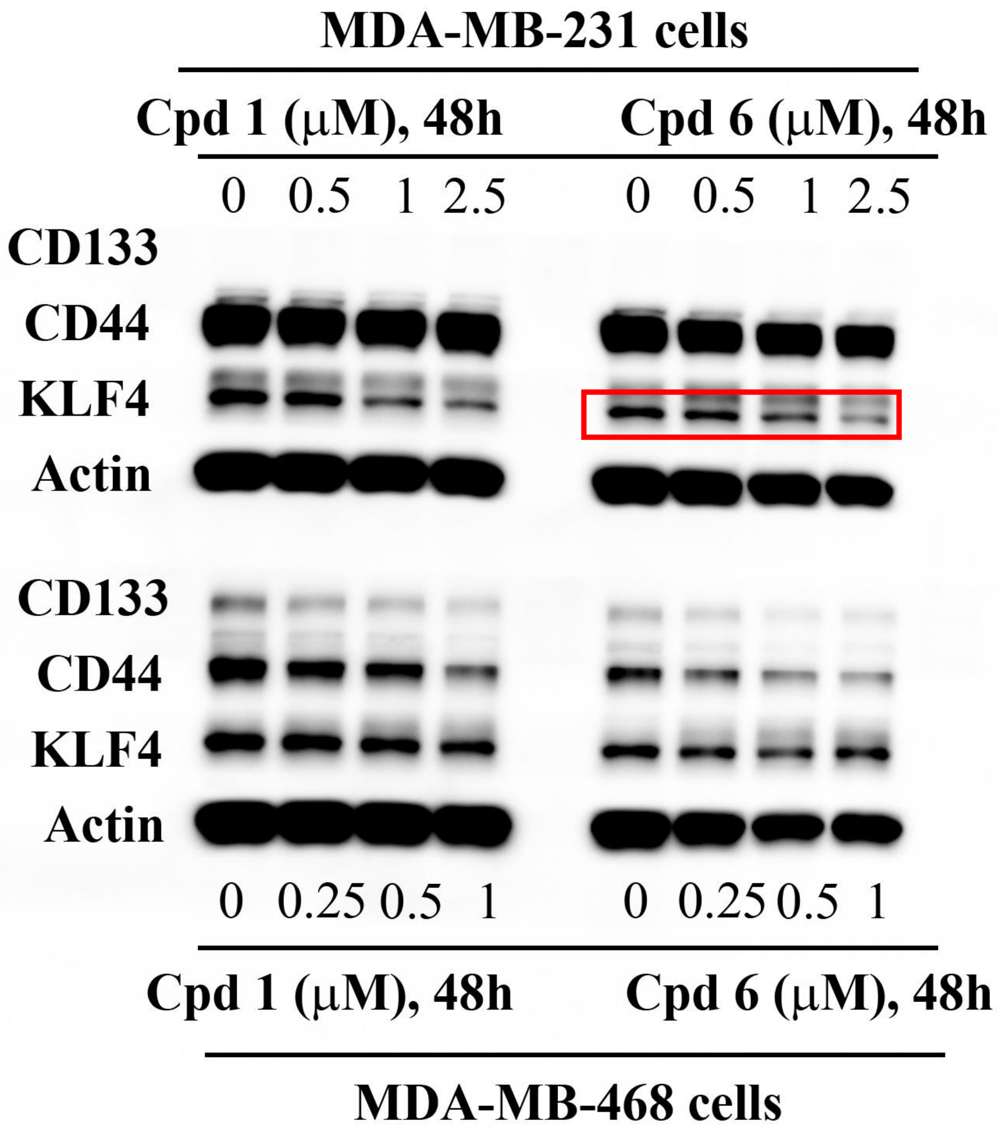
MDA-MB-231 cells
Cpd 1 (μM), 48h
Cpd 6 (μM), 48h
0 0.5 1 2.5
0 0.5 1 2.5
CD133
CD44
KLF4
Actin
CD133
CD44
KLF4
Actin
0 0.25 0.5 1
0 0.25 0.5 1
Cpd 1 (μM), 48h
Cpd 6 (μM), 48h
MDA-MB-468 cells

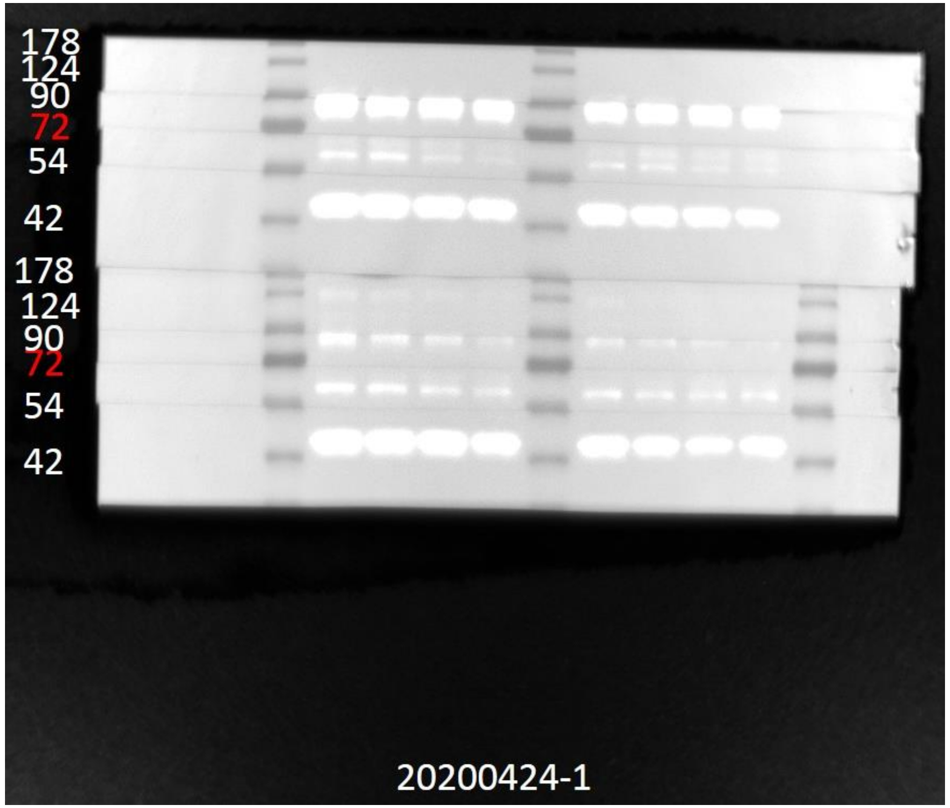
178
124
90
72
54
42
178
124
90
72
54
42
20200424-1

Fig. 4E (right)

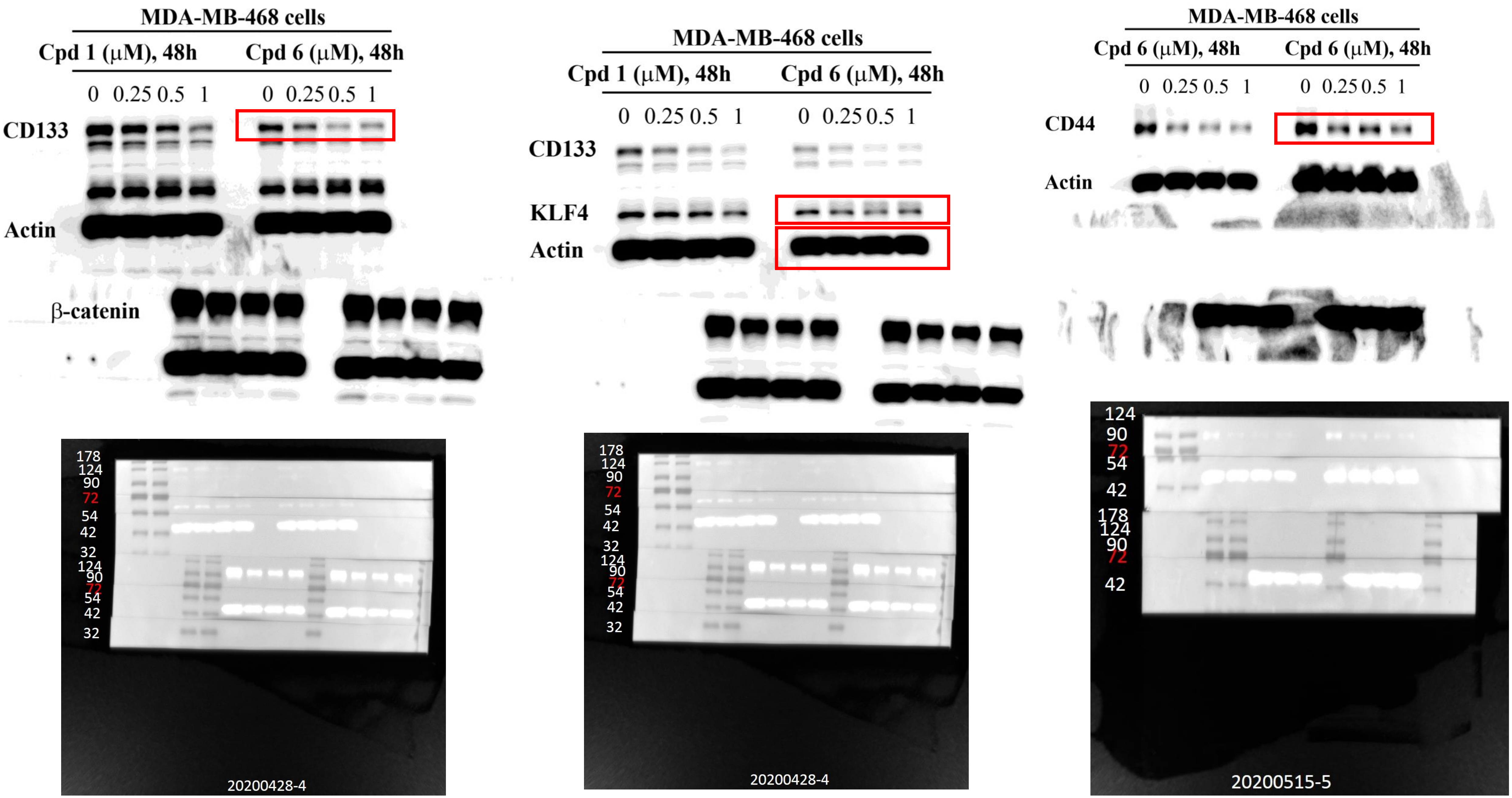

MDA-MB-468 cells
Cpd 1 (μM), 48h
Cpd 6 (μM), 48h
0 0.25 0.5 1
0 0.25 0.5 1
CD133
Actin
β-catenin
178
124
90
72
54
42
32
124
90
72
54
42
32
20200428-4
MDA-MB-468 cells
Cpd 1 (μM), 48h
Cpd 6 (μM), 48h
0 0.25 0.5 1
0 0.25 0.5 1
CD133
KLF4
Actin
178
124
90
72
54
42
32
124
90
72
54
42
32
20200428-4
MDA-MB-468 cells
Cpd 6 (μM), 48h
Cpd 6 (μM), 48h
0 0.25 0.5 1
0 0.25 0.5 1
CD44
Actin
124
90
72
54
42
178
124
90
72
42
20200515-5

Fig. 4F

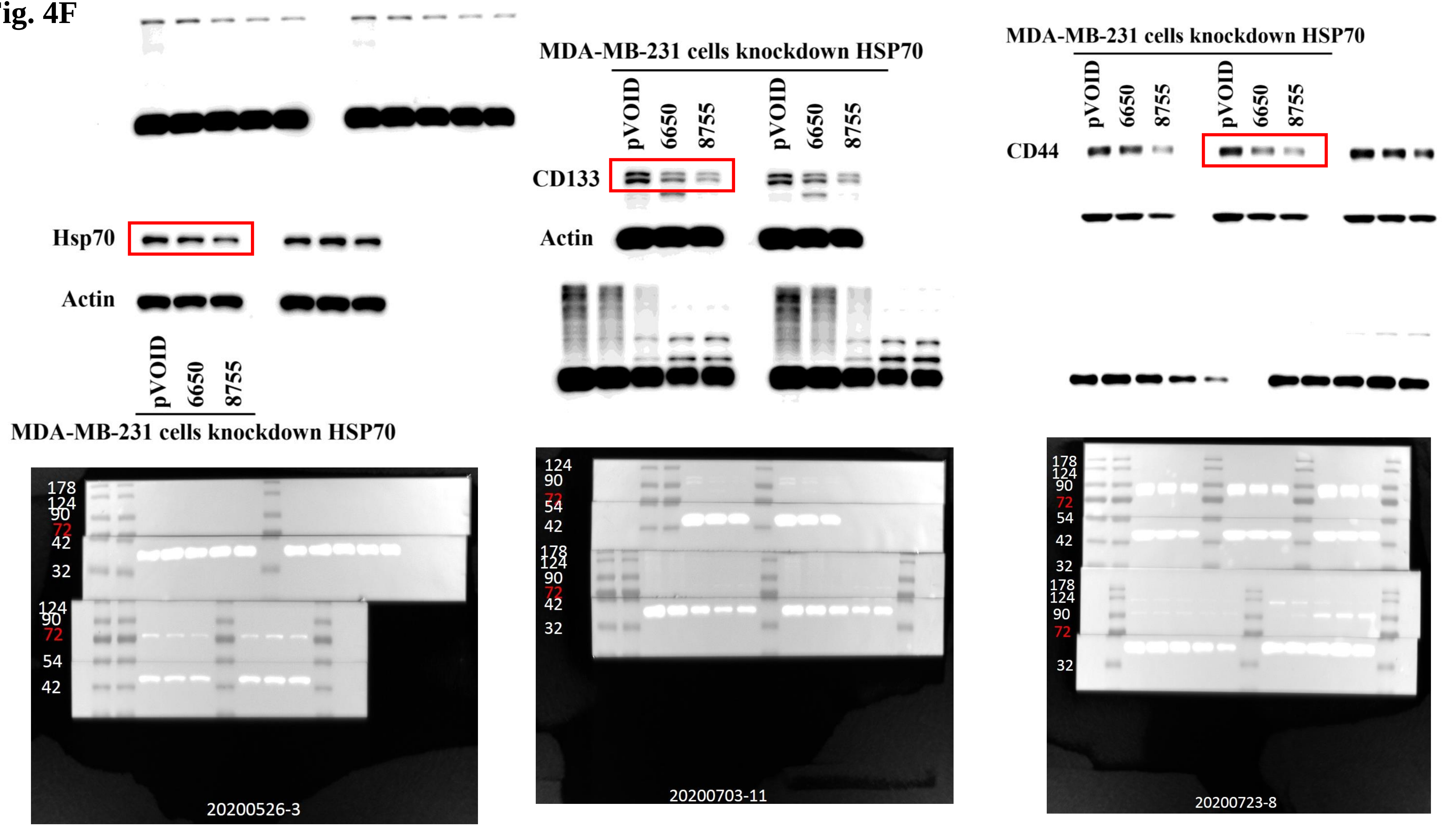

Hsp70
Actin
pVOID
6650
8755
MDA-MB-231 cells knockdown HSP70
MDA-MB-231 cells knockdown HSP70
pVOID
6650
8755
pVOID
6650
8755
CD133
Actin
MDA-MB-231 cells knockdown HSP70
pVOID
6650
8755
pVOID
6650
8755
CD44
178
124
90
72
42
32
124
90
72
54
42
20200526-3
124
90
72
54
42
178
124
90
72
42
32
20200703-11
178
124
90
72
54
42
32
178
124
90
72
32
20200723-8

Fig. 4F

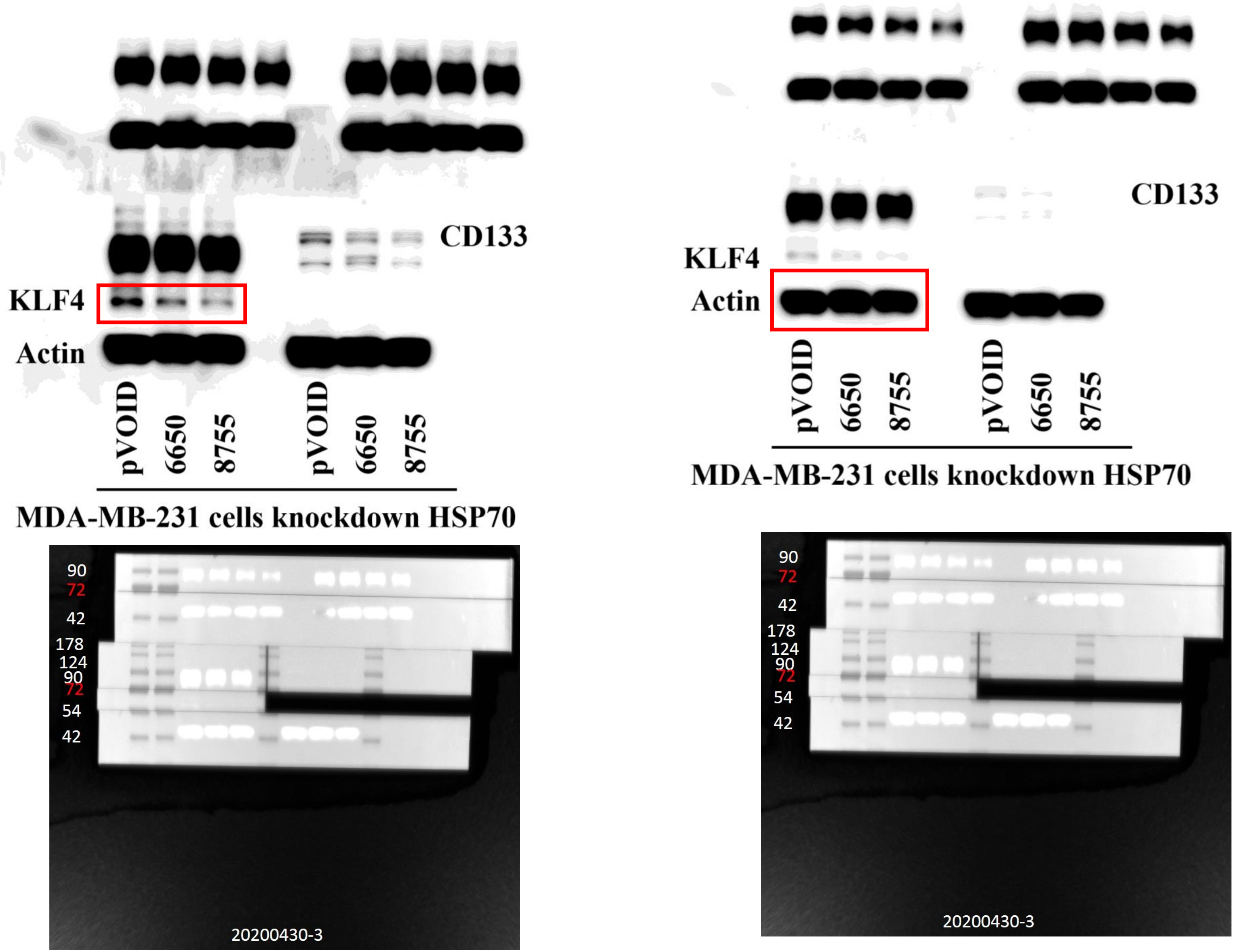

CD133
KLF4
Actin
pVOID
6650
8755
pVOID
6650
8755
MDA-MB-231 cells knockdown HSP70
90
72
42
178
124
90
72
54
42
20200430-3
CD133
KLF4
Actin
pVOID
6650
8755
pVOID
6650
8755
MDA-MB-231 cells knockdown HSP70
90
72
42
178
124
90
72
54
42
20200430-3

Fig. 5A

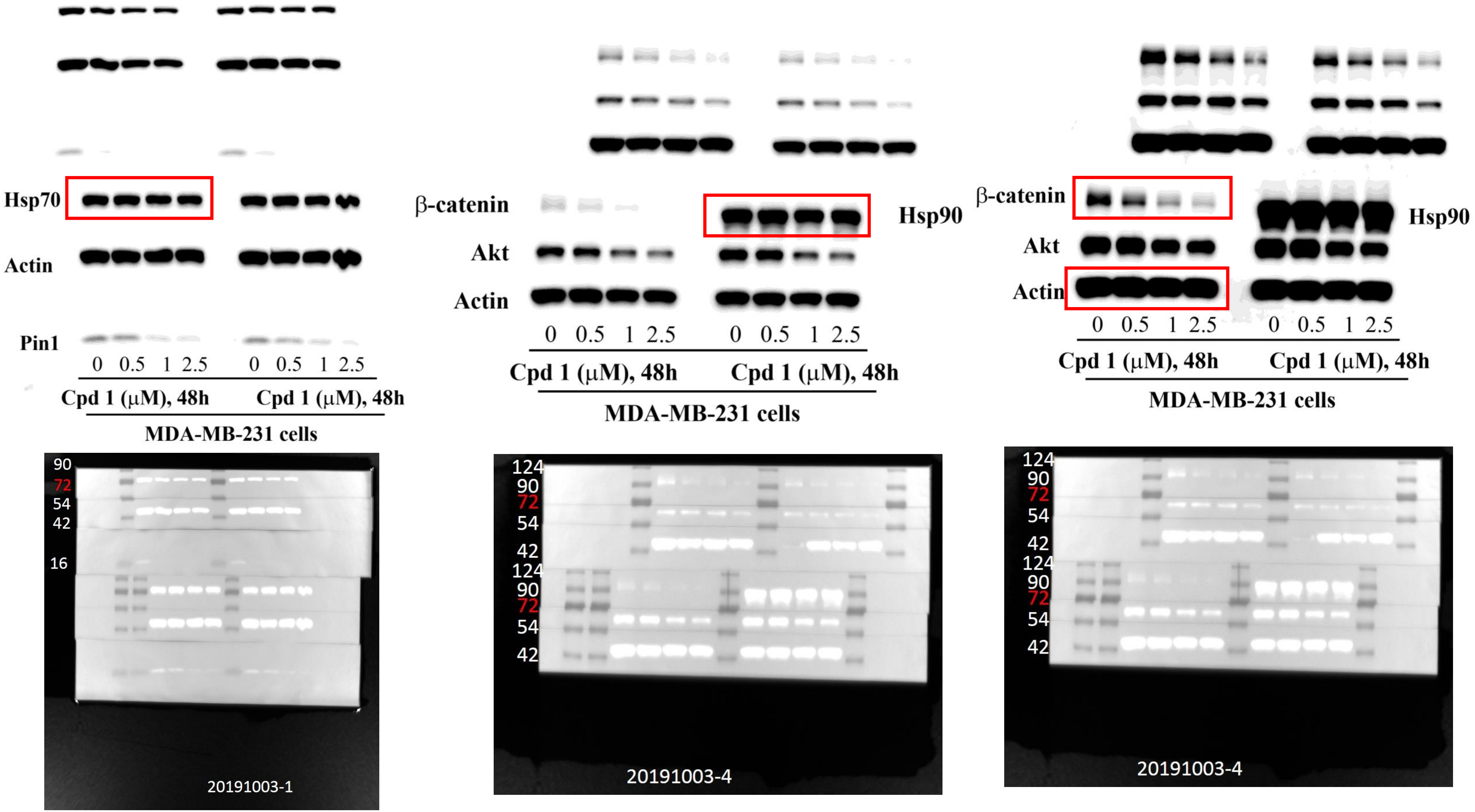
Hsp70
Actin
Pin1
0 0.5 1 2.5 0 0.5 1 2.5
Cpd 1 (μM), 48h Cpd 1 (μM), 48h
MDA-MB-231 cells
β-catenin
Akt
Actin
Hsp90
0 0.5 1 2.5 0 0.5 1 2.5
Cpd 1 (μM), 48h Cpd 1 (μM), 48h
MDA-MB-231 cells
β-catenin
Akt
Actin
Hsp90
0 0.5 1 2.5 0 0.5 1 2.5
Cpd 1 (μM), 48h Cpd 1 (μM), 48h
MDA-MB-231 cells
90
72
54
42
16
20191003-1
124
90
72
54
42
20191003-4
124
90
72
54
42
20191003-4

Fig. 5A

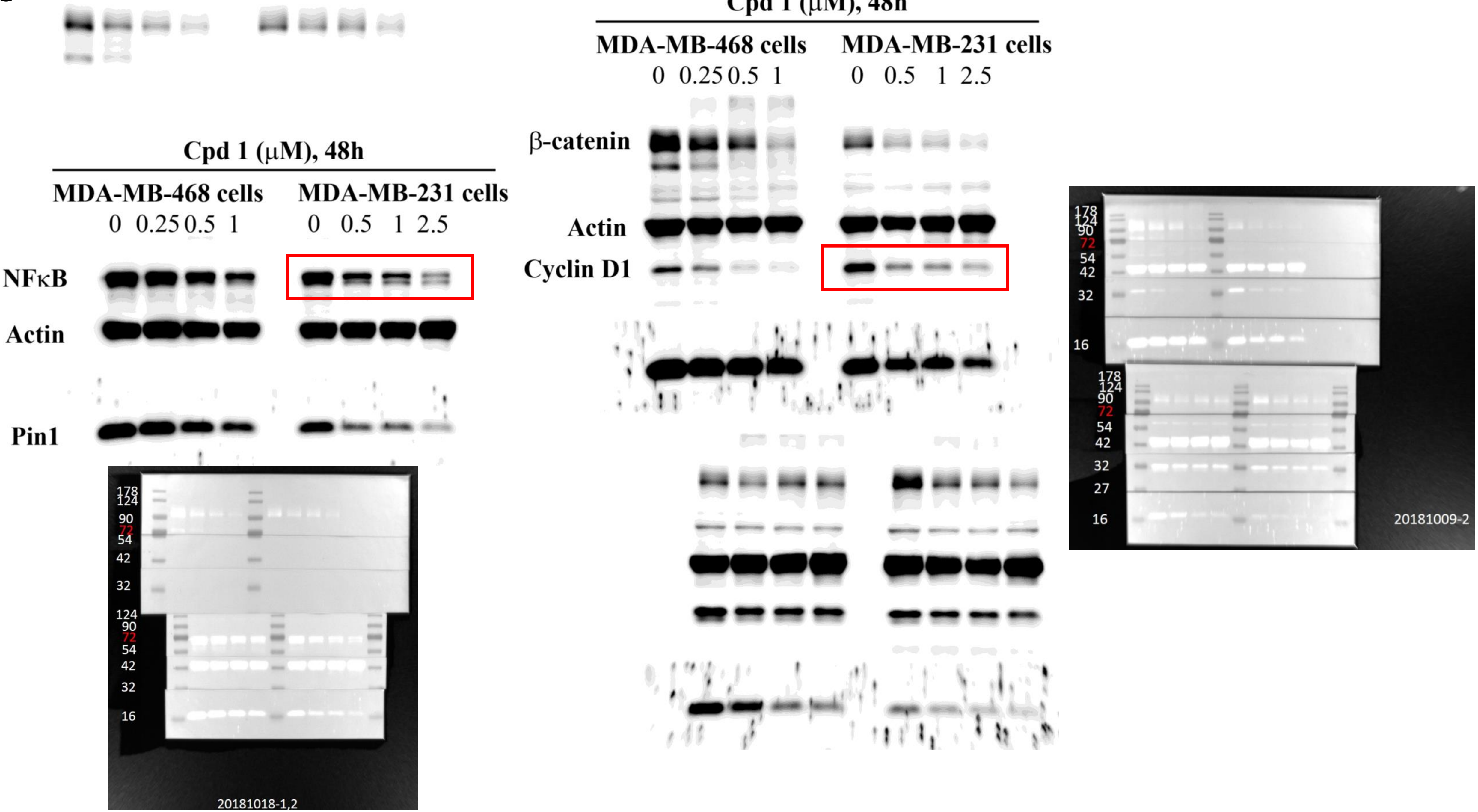
Cpd 1 (μM), 48h
MDA-MB-468 cells
MDA-MB-231 cells
0 0.25 0.5 1
0 0.5 1 2.5
NFκB
Actin
Pin1
178
124
90
72
54
42
32
16
20181018-1,2
Cpd 1 (μM), 48h
MDA-MB-468 cells
MDA-MB-231 cells
0 0.25 0.5 1
0 0.5 1 2.5
β-catenin
Actin
Cyclin D1
178
124
90
72
54
42
32
27
16
20181009-2

Fig. 5A

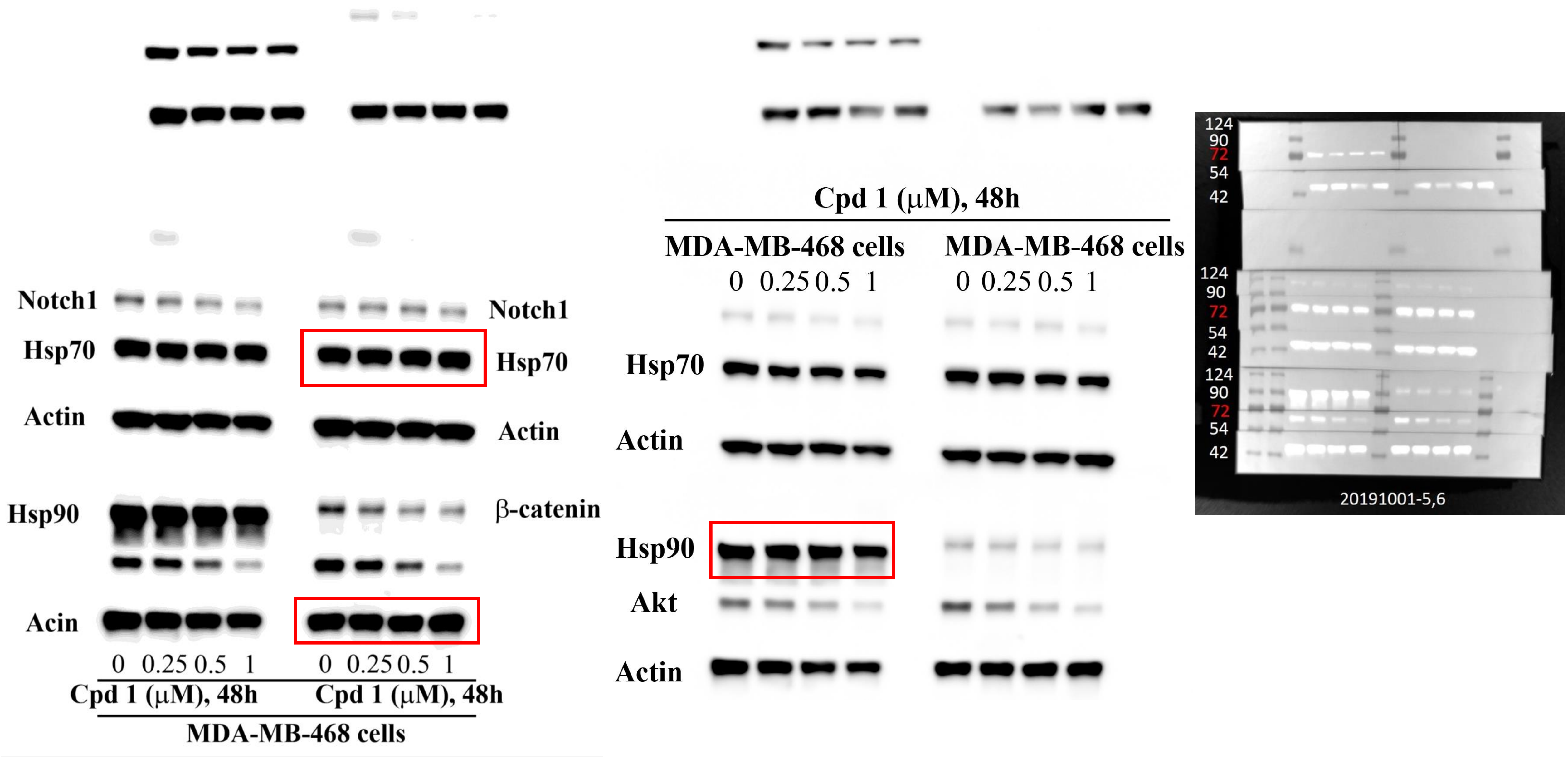

Notch1
Hsp70
Actin
Hsp90
Acin
0 0.25 0.5 1
Cpd 1 (μM), 48h
Notch1
Hsp70
Actin
β-catenin
0 0.25 0.5 1
Cpd 1 (μM), 48h
MDA-MB-468 cells
Cpd 1 (μM), 48h
MDA-MB-468 cells
0 0.25 0.5 1
MDA-MB-468 cells
0 0.25 0.5 1
Hsp70
Actin
Hsp90
Akt
Actin
124
90
72
54
42
20191001-5,6

Fig. 5A

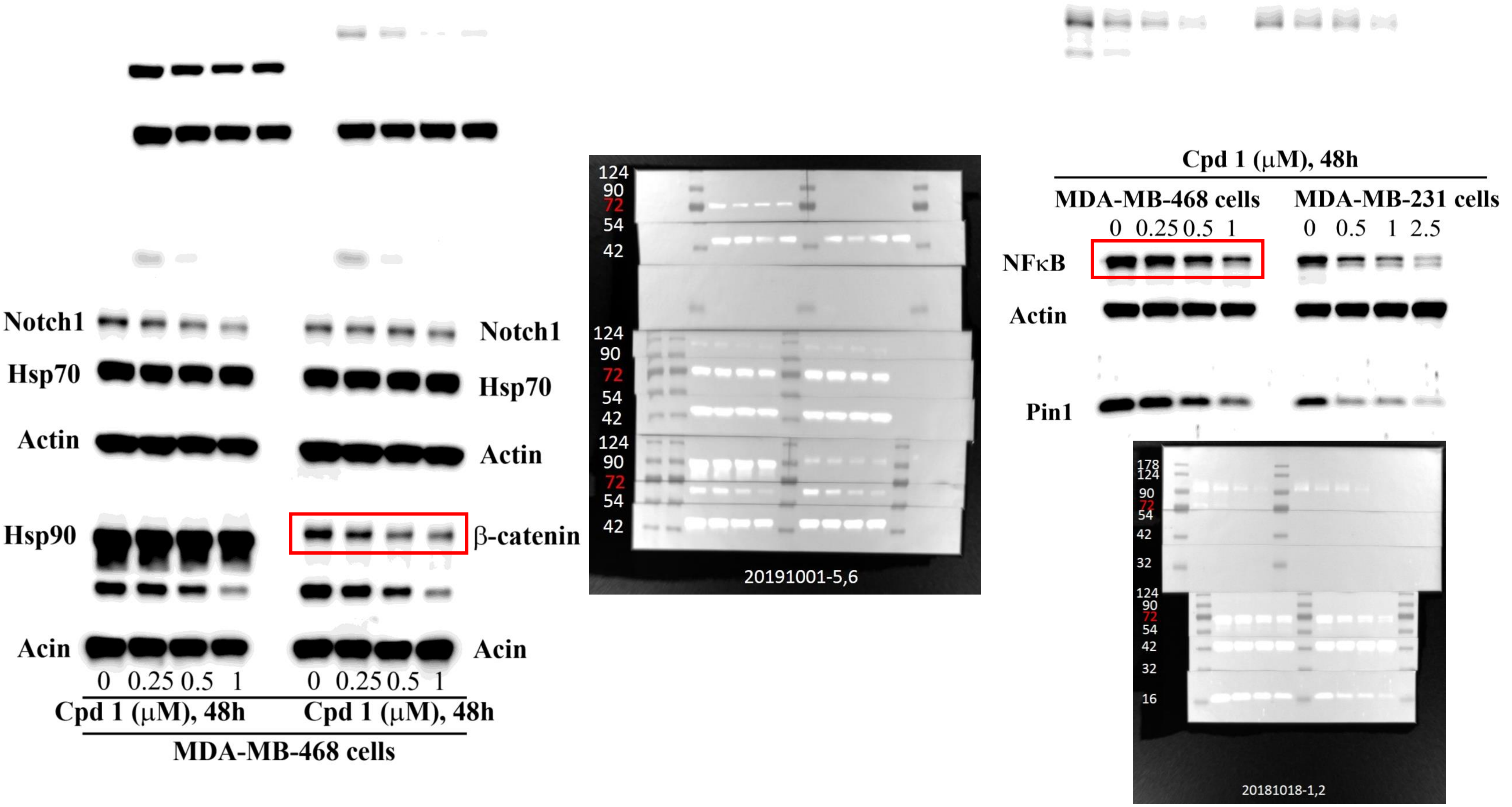

Notch1
Hsp70
Actin
Hsp90
Acin
0 0.25 0.5 1
Cpd 1 (μM), 48h
Notch1
Hsp70
Actin
β-catenin
Acin
0 0.25 0.5 1
Cpd 1 (μM), 48h
MDA-MB-468 cells
124
90
72
54
42
124
90
72
54
42
124
90
72
54
42
20191001-5,6
Cpd 1 (μM), 48h
MDA-MB-468 cells
MDA-MB-231 cells
0 0.25 0.5 1
0 0.5 1 2.5
NFκB
Actin
Pin1
178
124
90
72
54
42
32
124
90
72
54
42
32
16
20181018-1,2

Fig. 5A

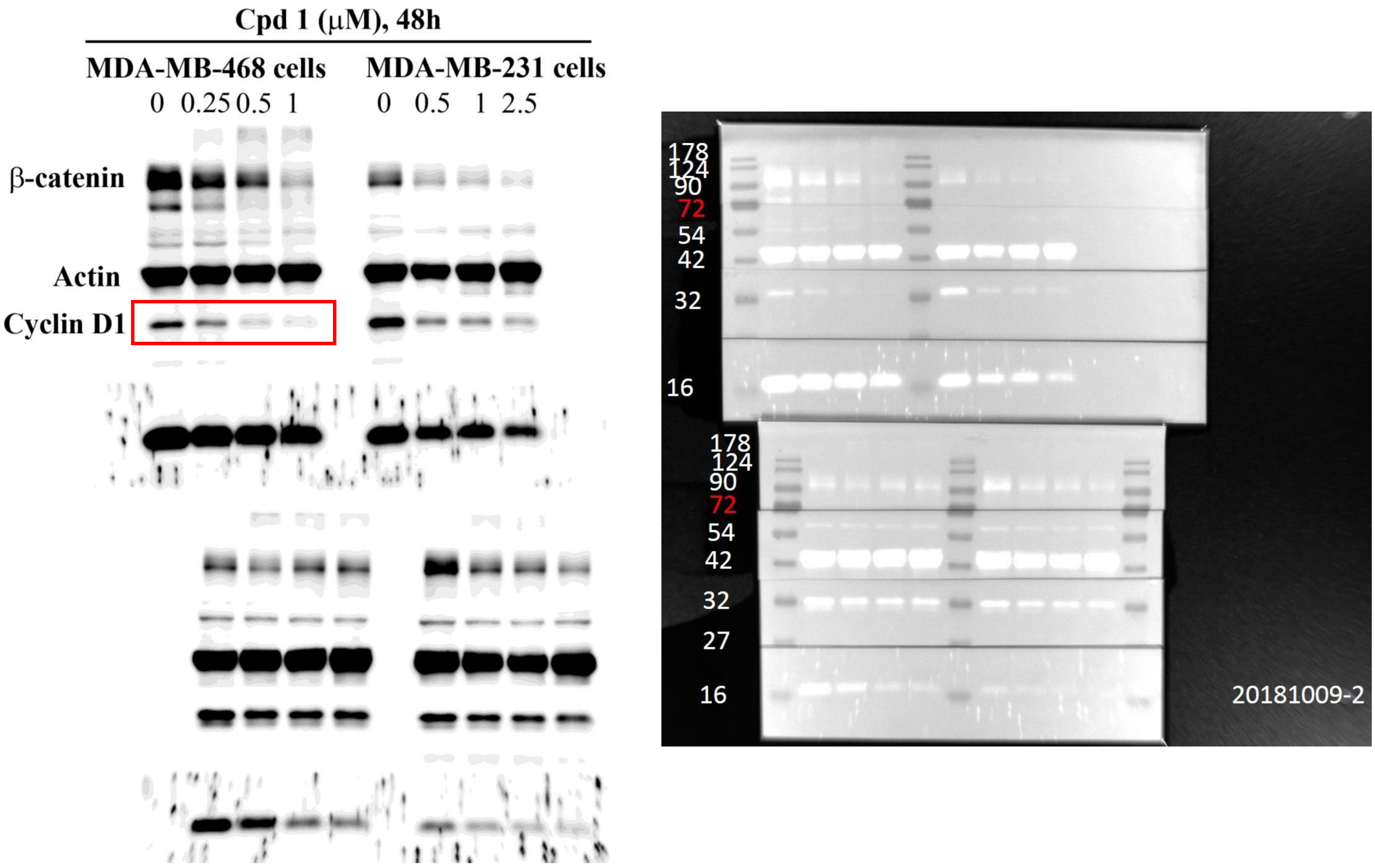
Cpd 1 (μM), 48h
MDA-MB-468 cells
MDA-MB-231 cells
0 0.25 0.5 1
0 0.5 1 2.5
β-catenin
Actin
Cyclin D1
178
124
90
72
54
42
32
16
178
124
90
72
54
42
32
27
16
20181009-2

Fig. 5A

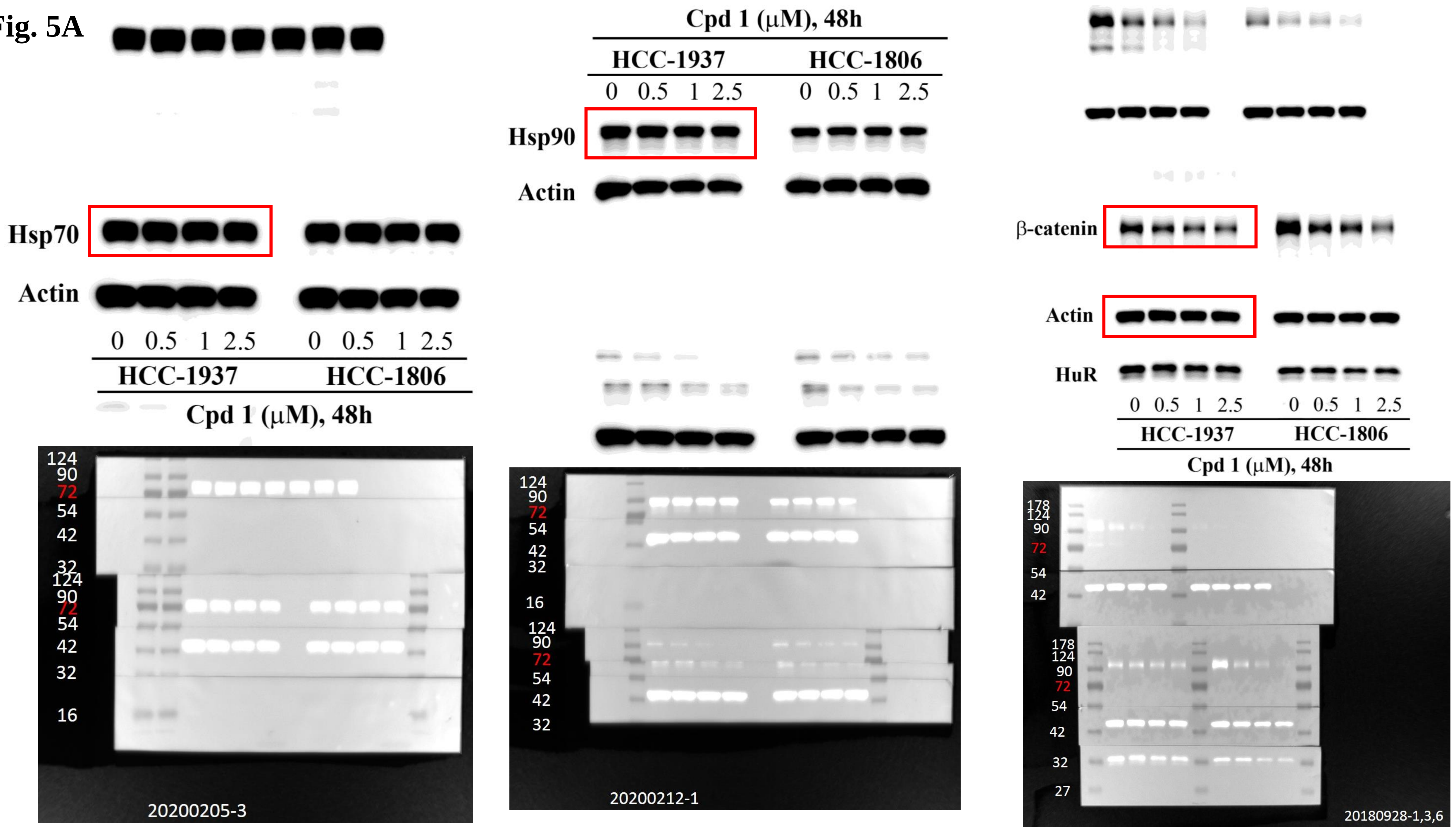
Hsp70
Actin
0 0.5 1 2.5 0 0.5 1 2.5
HCC-1937
HCC-1806
Cpd 1 (μM), 48h
124
90
72
54
42
32
124
90
72
54
42
32
16
20200205-3
Cpd 1 (μM), 48h
HCC-1937
HCC-1806
0 0.5 1 2.5 0 0.5 1 2.5
Hsp90
Actin
124
90
72
54
42
32
16
124
90
72
54
42
32
20200212-1
β-catenin
Actin
HuR
0 0.5 1 2.5 0 0.5 1 2.5
HCC-1937
HCC-1806
Cpd 1 (μM), 48h
178
124
90
72
54
42
178
124
90
72
54
42
32
27
20180928-1,3,6

Fig. 5A

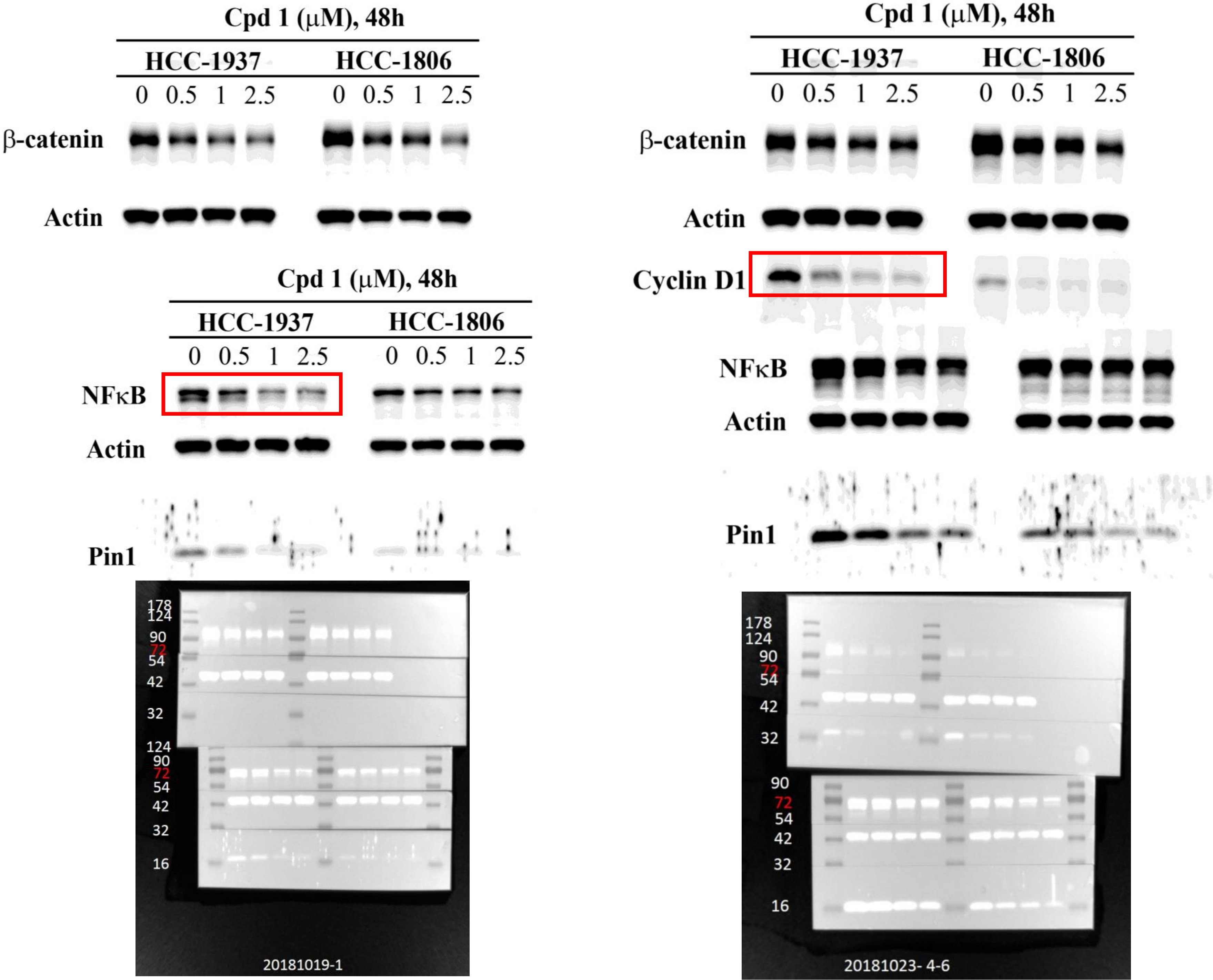

Cpd 1 (μM), 48h
HCC-1937
HCC-1806
0 0.5 1 2.5
0 0.5 1 2.5
β-catenin
Actin
Cyclin D1
NFκB
Actin
Pin1
178
124
90
72
54
42
32
16
20181019-1
20181023- 4-6

Fig. 5A

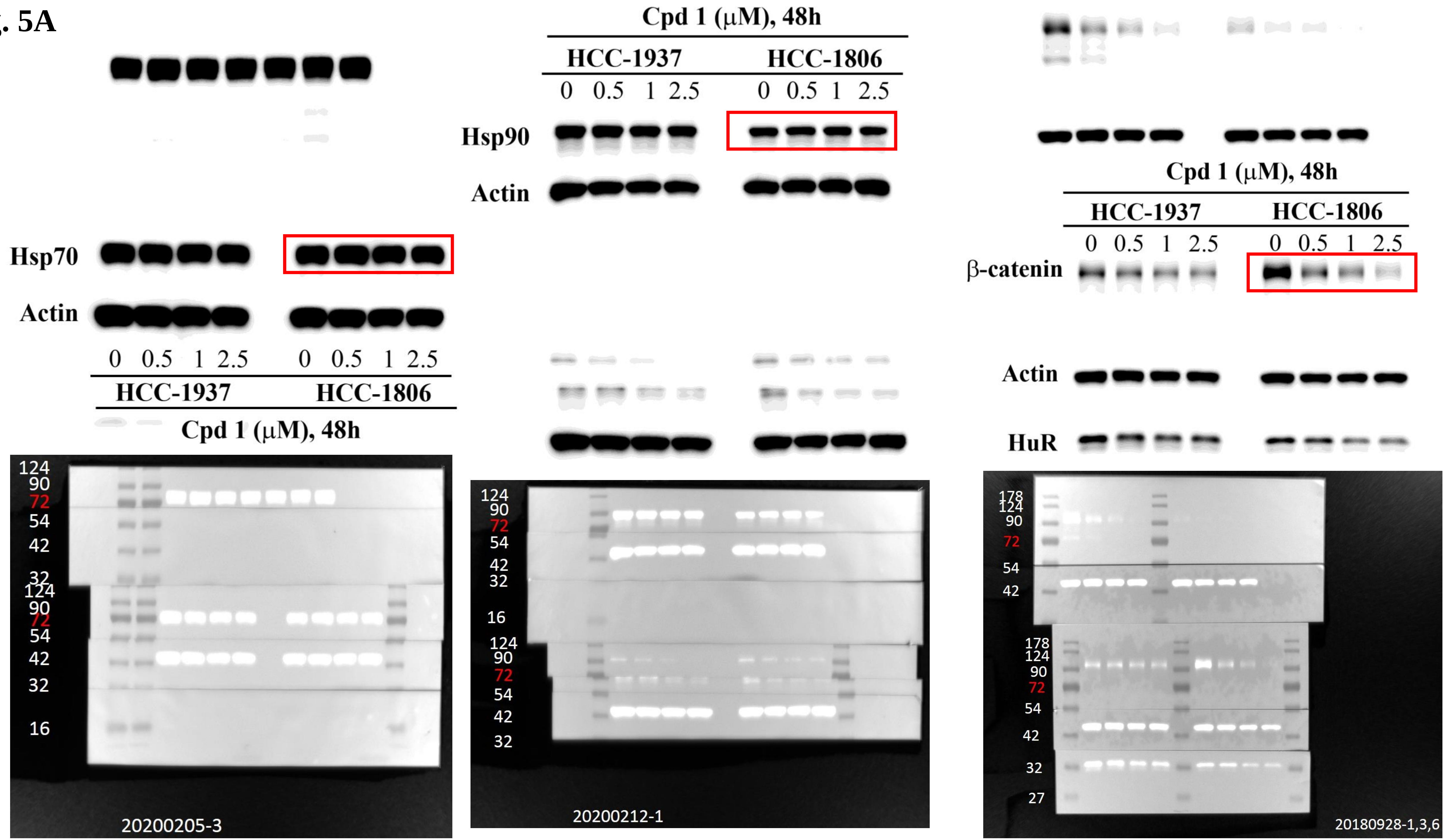

Cpd 1 (μM), 48h
HCC-1937
HCC-1806
0 0.5 1 2.5
0 0.5 1 2.5
Hsp90
Actin
Hsp70
Actin
0 0.5 1 2.5
0 0.5 1 2.5
HCC-1937
HCC-1806
Cpd 1 (μM), 48h
Cpd 1 (μM), 48h
HCC-1937
HCC-1806
0 0.5 1 2.5
0 0.5 1 2.5
β-catenin
Actin
HuR
20200205-3
20200212-1
20180928-1,3,6

Fig. 5A

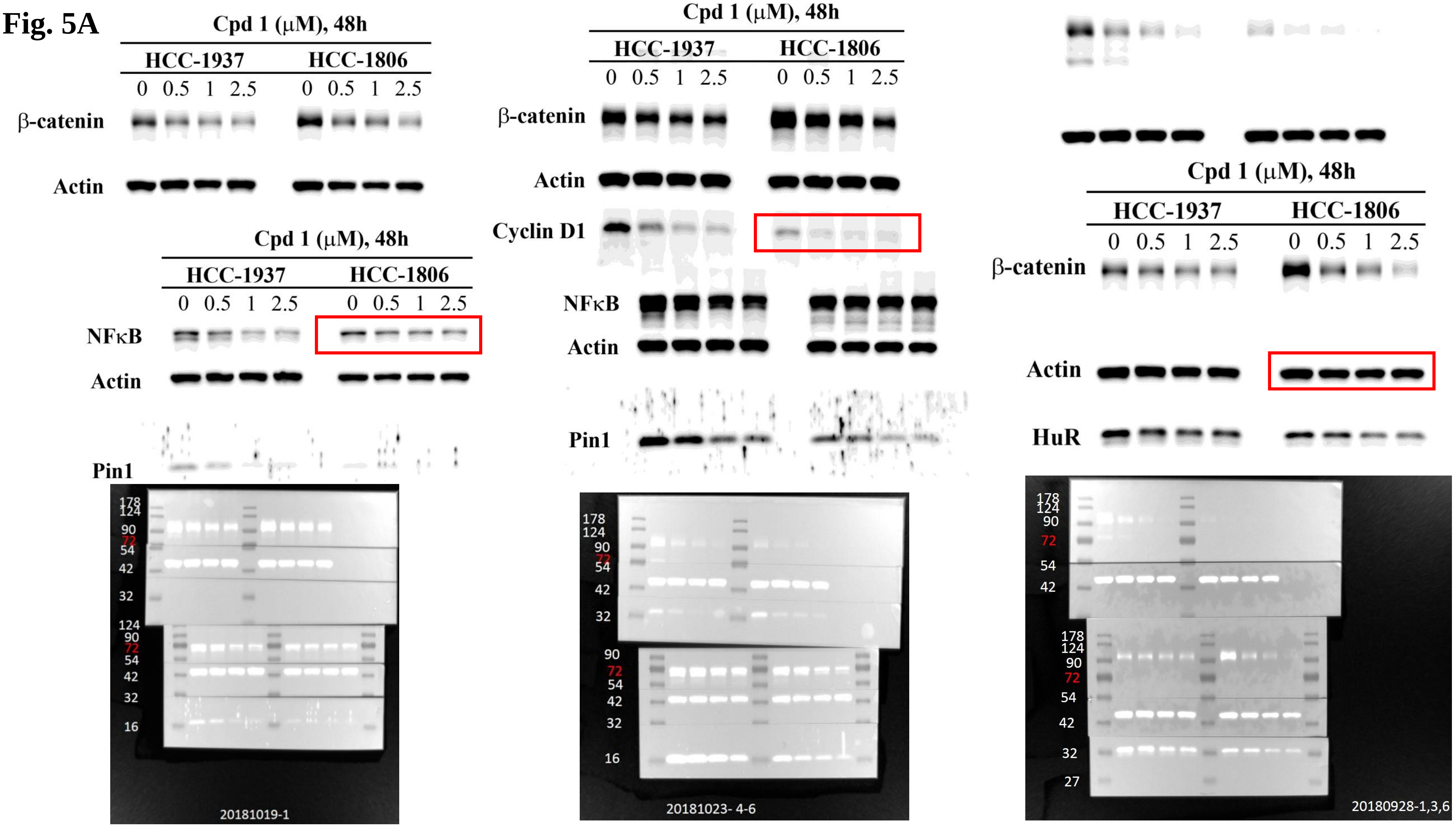
Cpd 1 (μM), 48h
HCC-1937
HCC-1806
0
0.5
1
2.5
β-catenin
Actin
NFκB
Pin1
Cyclin D1
HuR
178
124
90
72
54
42
32
16
27
20181019-1
20181023- 4-6
20180928-1,3,6

Fig. 5B

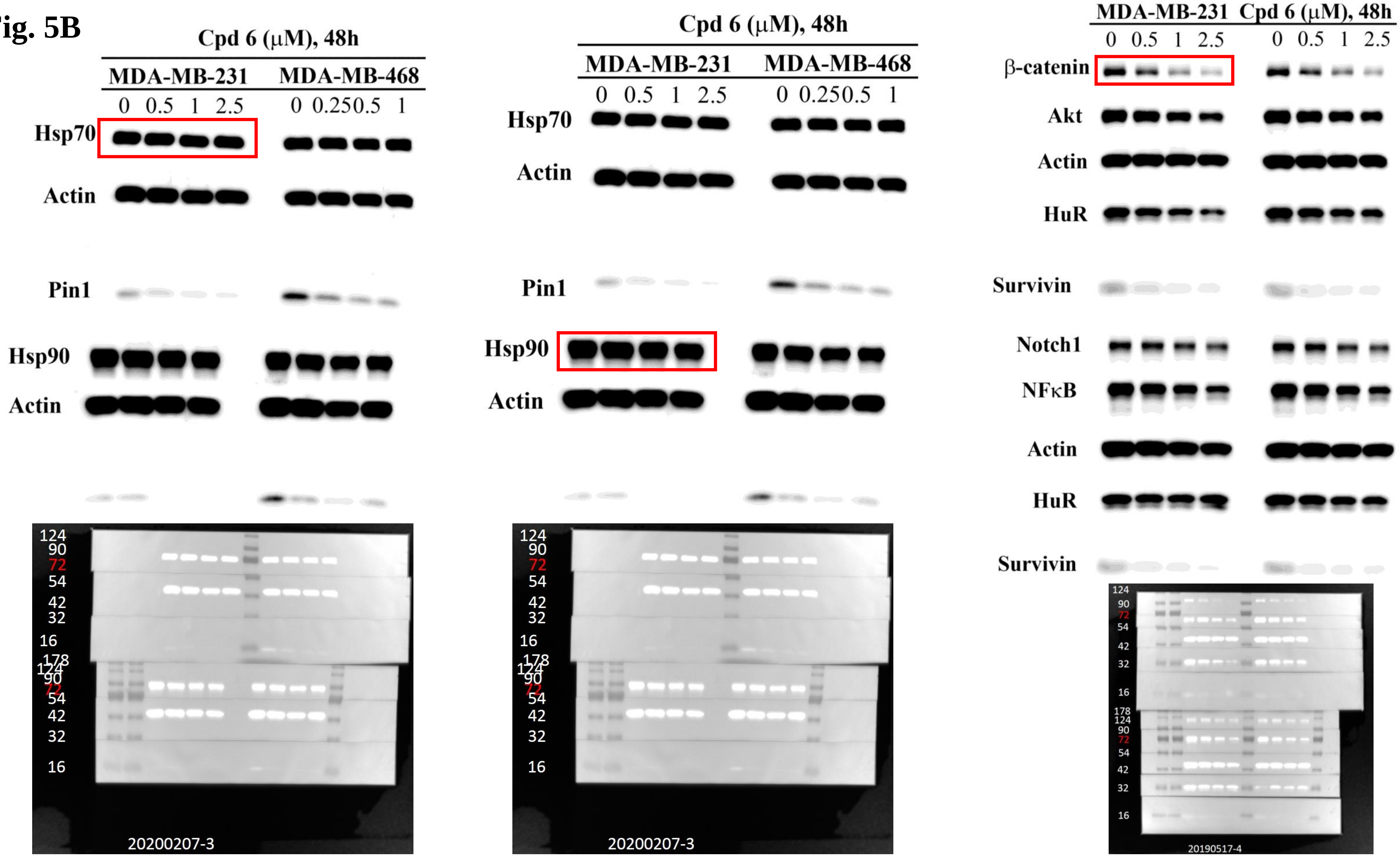

Cpd 6 (μM), 48h
MDA-MB-231
MDA-MB-468
0 0.5 1 2.5
0 0.25 0.5 1
Hsp70
Actin
Pin1
Hsp90
Actin
124
90
72
54
42
32
16
178
20200207-3
Cpd 6 (μM), 48h
MDA-MB-231
MDA-MB-468
0 0.5 1 2.5
0 0.25 0.5 1
Hsp70
Actin
Pin1
Hsp90
Actin
20200207-3
MDA-MB-231 Cpd 6 (μM), 48h
0 0.5 1 2.5
0 0.5 1 2.5
β-catenin
Akt
Actin
HuR
Survivin
Notch1
NFκB
Actin
HuR
Survivin
20190517-4

Fig. 5B

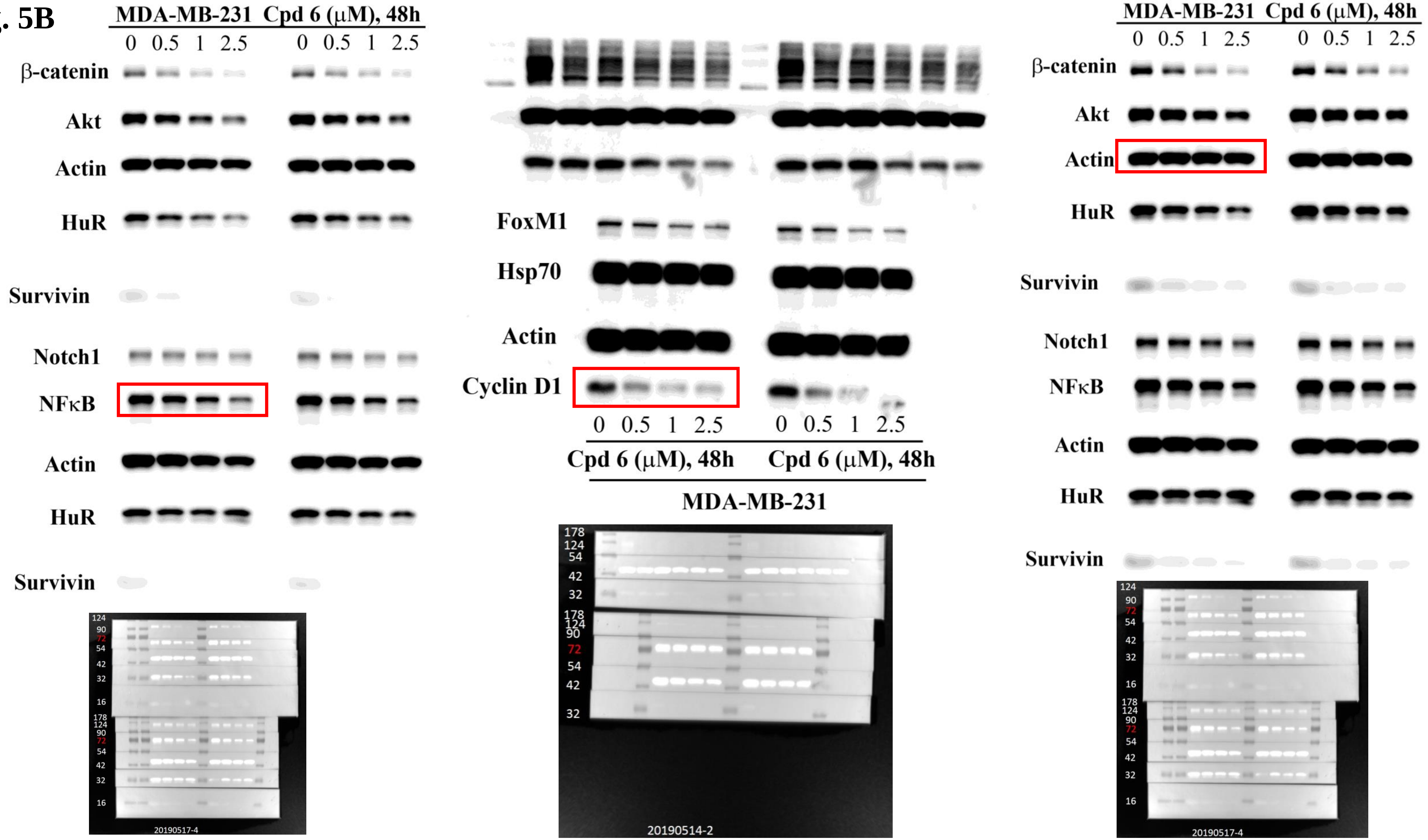

MDA-MB-231 Cpd 6 (μM), 48h
0 0.5 1 2.5 0 0.5 1 2.5
β-catenin
Akt
Actin
HuR
Survivin
Notch1
NFκB
Actin
HuR
Survivin
FoxM1
Hsp70
Actin
Cyclin D1
0 0.5 1 2.5 0 0.5 1 2.5
Cpd 6 (μM), 48h Cpd 6 (μM), 48h
MDA-MB-231
178
124
54
42
32
178
124
90
72
54
42
32
20190514-2
124
90
72
54
42
32
16
178
124
90
72
54
42
32
16
20190517-4
MDA-MB-231 Cpd 6 (μM), 48h
0 0.5 1 2.5 0 0.5 1 2.5
β-catenin
Akt
Actin
HuR
Survivin
Notch1
NFκB
Actin
HuR
Survivin
124
90
72
54
42
32
16
178
124
90
72
54
42
32
16
20190517-4

Fig. 5B

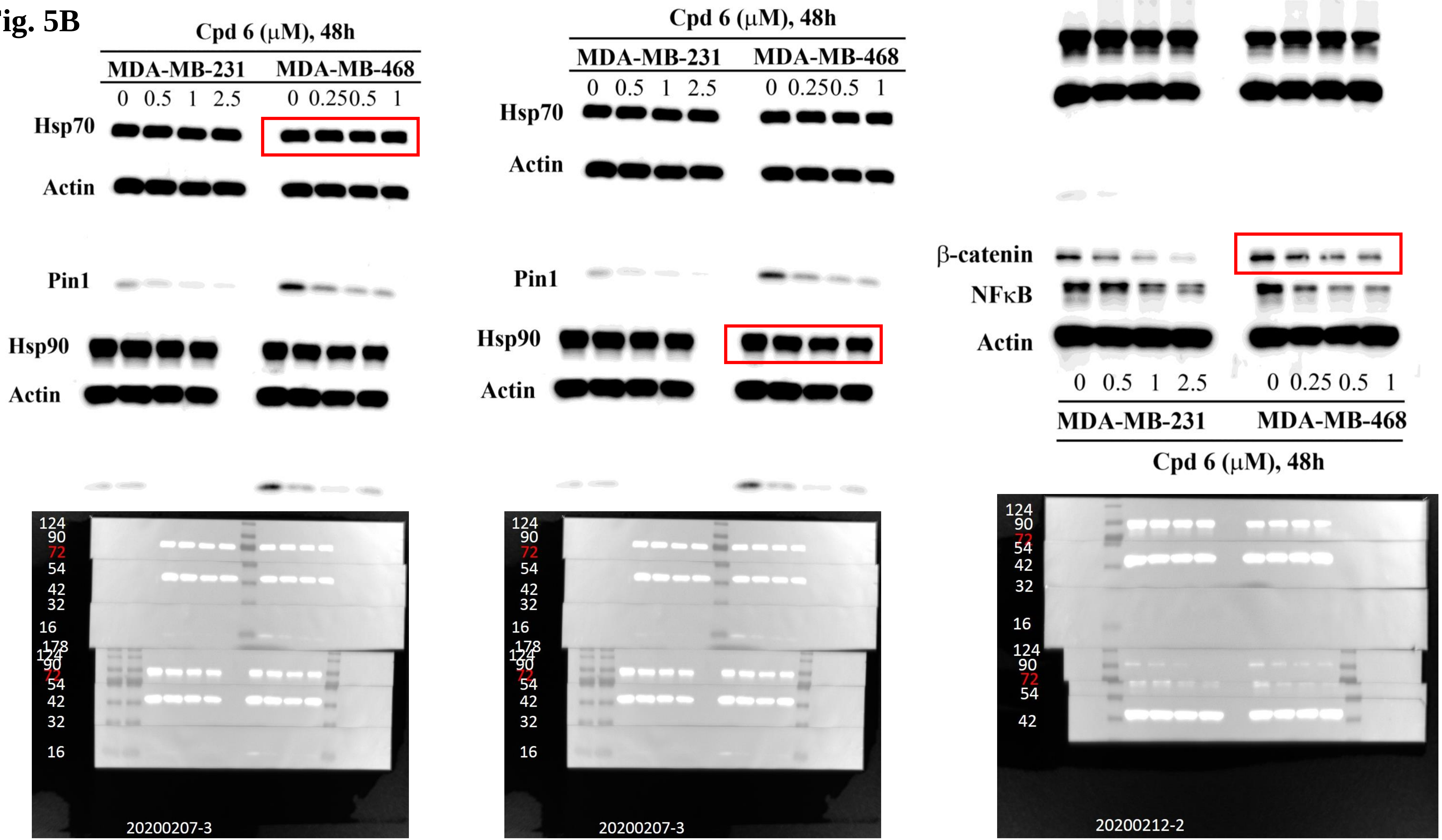

Cpd 6 (μM), 48h
MDA-MB-231
MDA-MB-468
0 0.5 1 2.5
0 0.25 0.5 1
Hsp70
Actin
Pin1
Hsp90
Actin
Cpd 6 (μM), 48h
MDA-MB-231
MDA-MB-468
0 0.5 1 2.5
0 0.25 0.5 1
Hsp70
Actin
Pin1
Hsp90
Actin
β-catenin
NFκB
Actin
0 0.5 1 2.5
0 0.25 0.5 1
MDA-MB-231
MDA-MB-468
Cpd 6 (μM), 48h
124
90
72
54
42
32
16
178
124
90
72
54
42
32
16
20200207-3
124
90
72
54
42
32
16
178
124
90
72
54
42
32
16
20200207-3
124
90
72
54
42
32
16
124
90
72
54
42
20200212-2

Fig. 5B

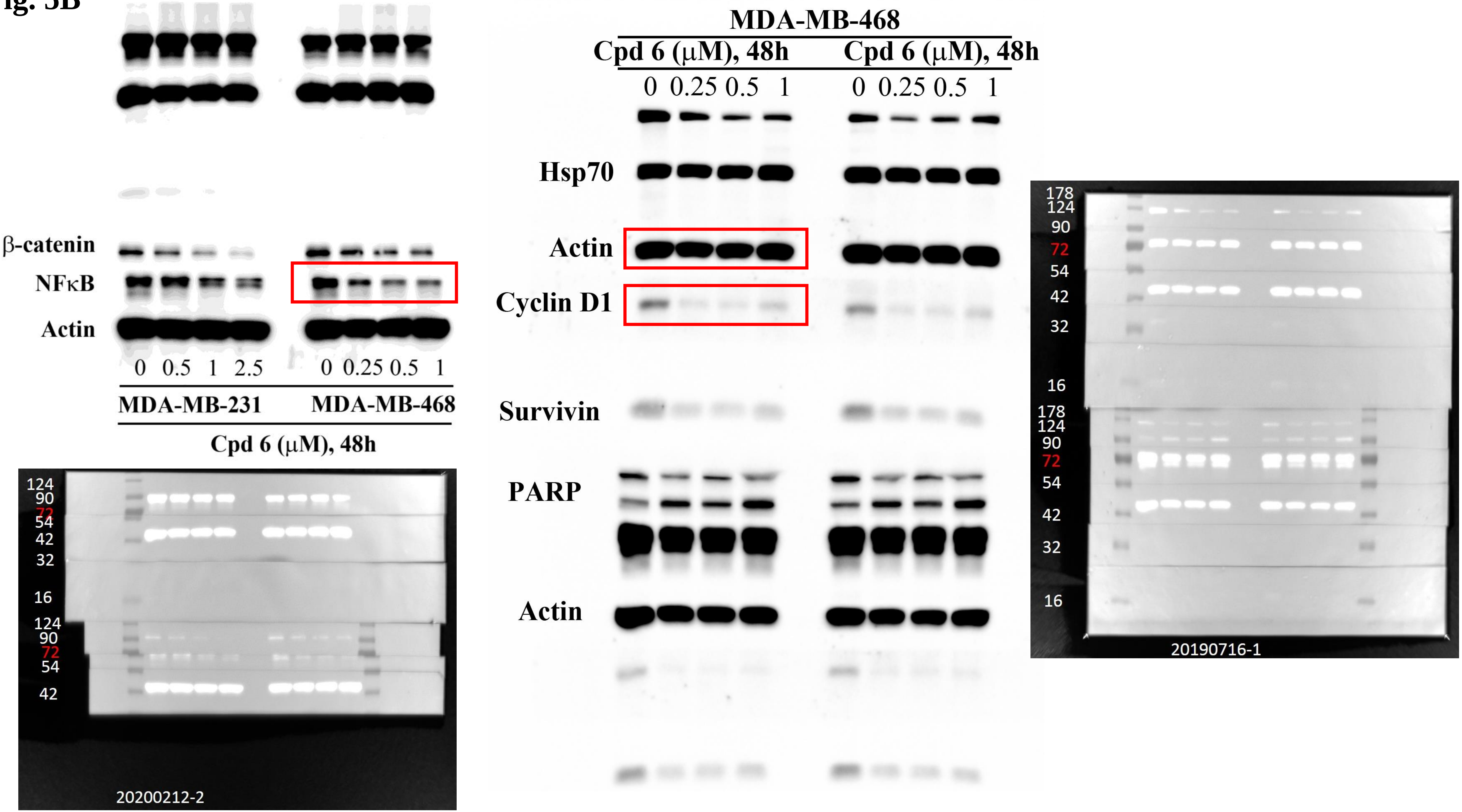

β-catenin
NFκB
Actin
0 0.5 1 2.5
0 0.25 0.5 1
MDA-MB-231
MDA-MB-468
Cpd 6 (μM), 48h
124
90
72
54
42
32
16
124
90
72
54
42
20200212-2
MDA-MB-468
Cpd 6 (μM), 48h
Cpd 6 (μM), 48h
0 0.25 0.5 1
0 0.25 0.5 1
Hsp70
Actin
Cyclin D1
Survivin
PARP
Actin
178
124
90
72
54
42
32
16
178
124
90
72
54
42
32
16
20190716-1

Fig. 5B

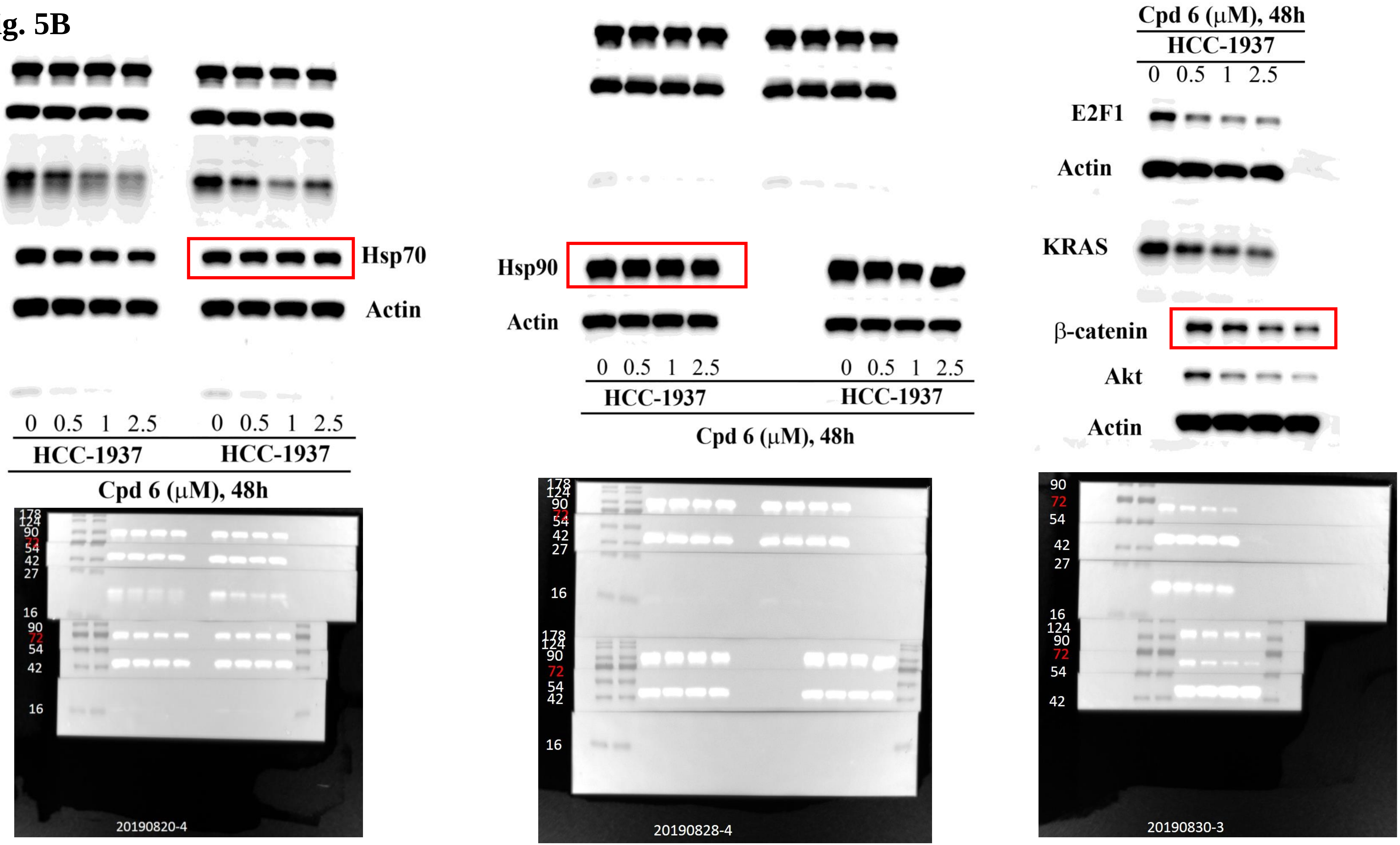
Hsp70
Actin
0 0.5 1 2.5
0 0.5 1 2.5
HCC-1937
HCC-1937
Cpd 6 (μM), 48h
178
124
90
72
54
42
27
16
20190820-4
Hsp90
Actin
0 0.5 1 2.5
0 0.5 1 2.5
HCC-1937
HCC-1937
Cpd 6 (μM), 48h
20190828-4
Cpd 6 (μM), 48h
HCC-1937
0 0.5 1 2.5
E2F1
Actin
KRAS
β-catenin
Akt
Actin
20190830-3

Fig. 5B

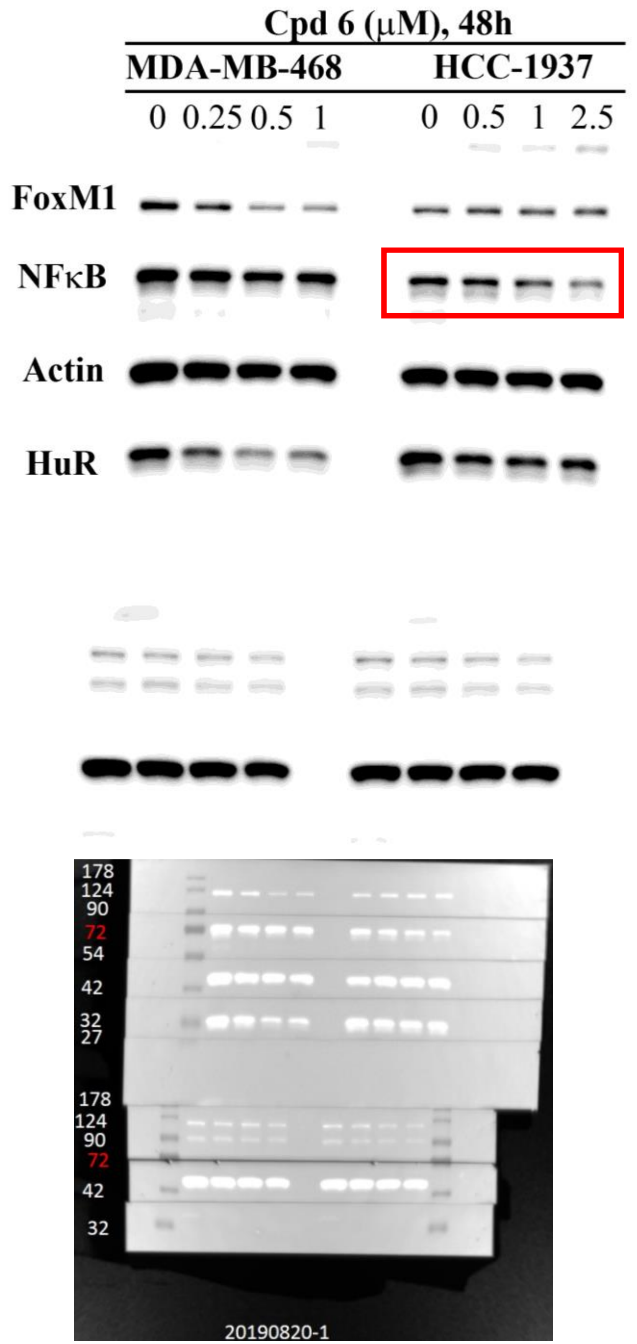
Cpd 6 (μM), 48h
MDA-MB-468
HCC-1937
0 0.25 0.5 1
0 0.5 1 2.5
FoxM1
NFκB
Actin
HuR
178
124
90
72
54
42
32
27
178
124
90
72
42
32
20190820-1

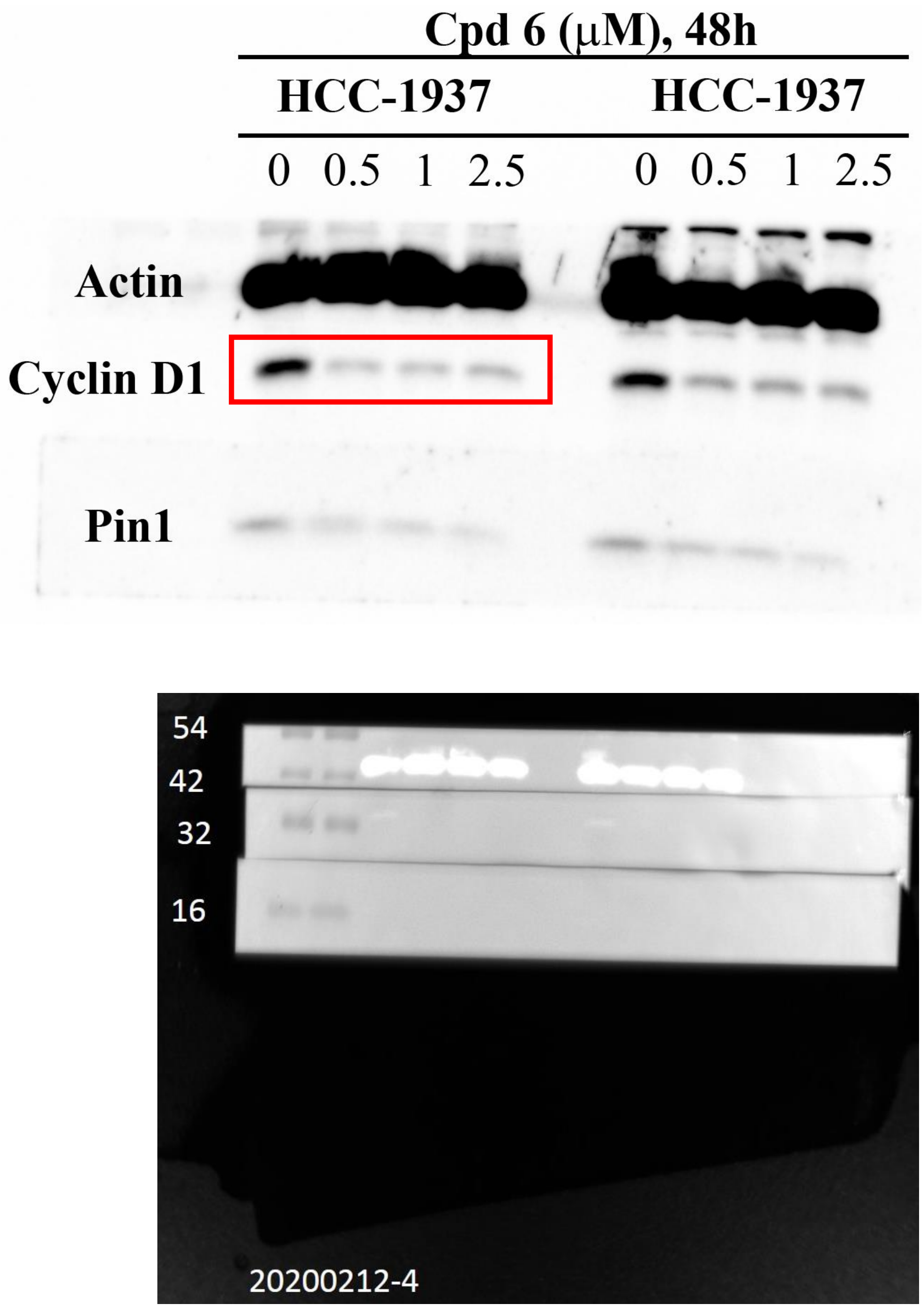
Cpd 6 (μM), 48h
HCC-1937
HCC-1937
0 0.5 1 2.5
0 0.5 1 2.5
Actin
Cyclin D1
Pin1
54
42
32
16
20200212-4

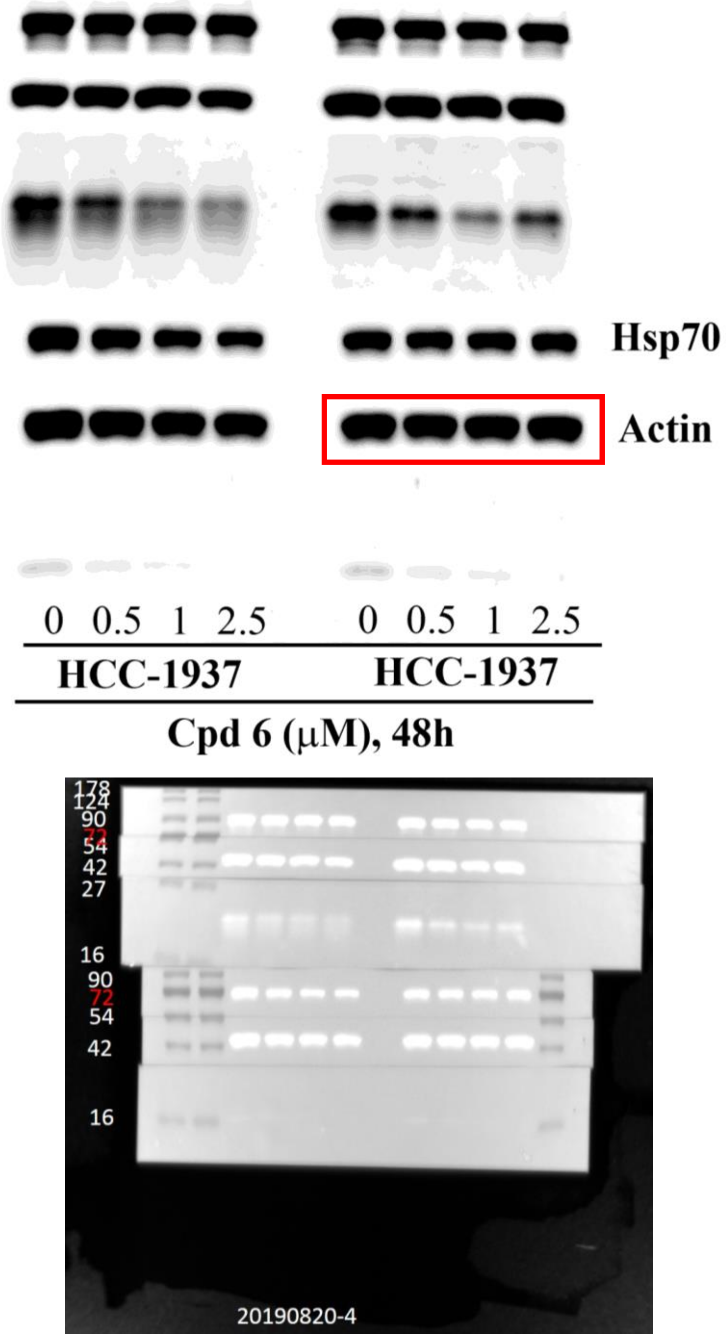
Hsp70
Actin
0 0.5 1 2.5
0 0.5 1 2.5
HCC-1937
HCC-1937
Cpd 6 (μM), 48h
178
124
90
72
54
42
27
16
90
72
54
42
16
20190820-4

Fig. 5B

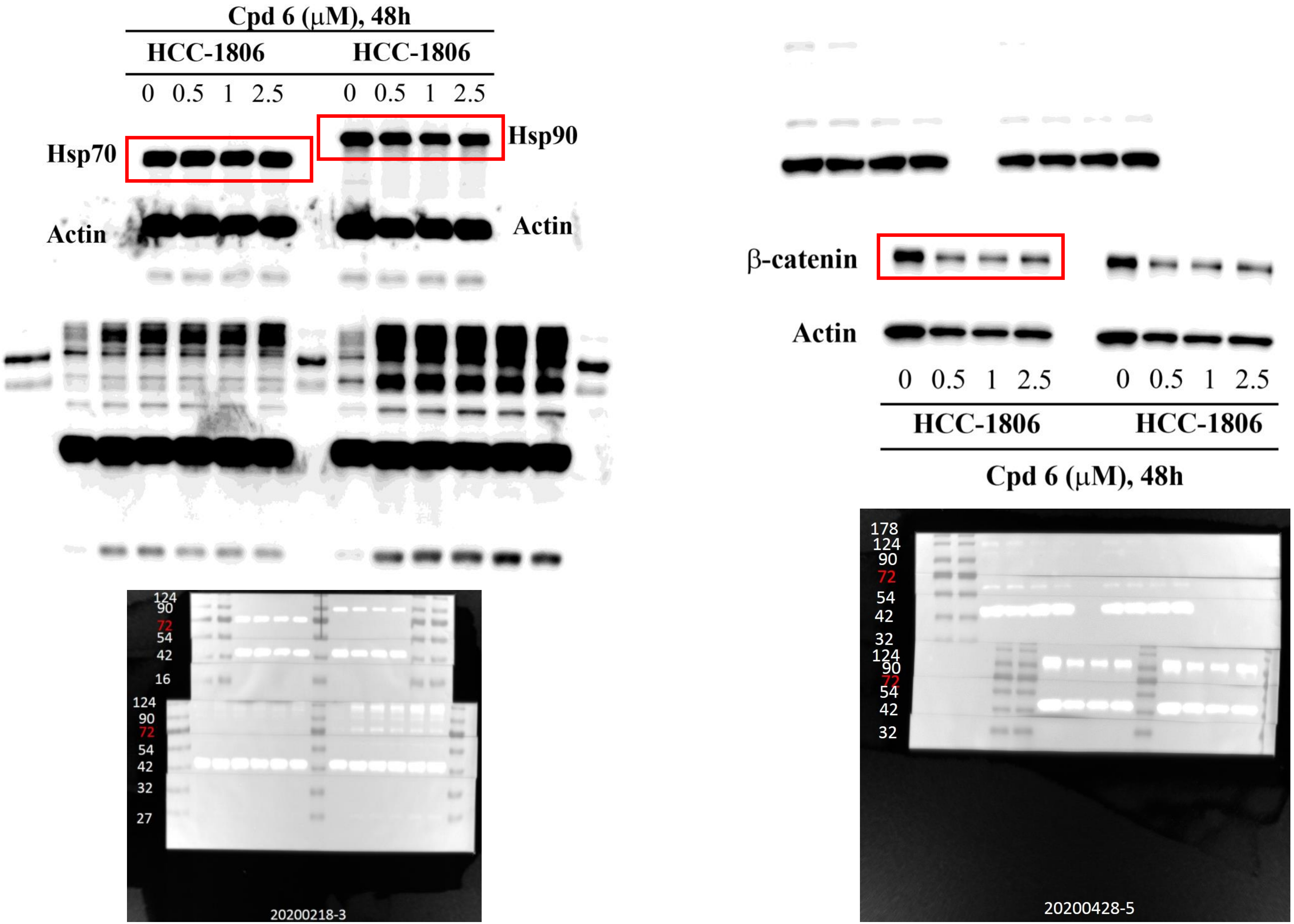
Cpd 6 (μM), 48h
HCC-1806
HCC-1806
0 0.5 1 2.5
0 0.5 1 2.5
Hsp70
Hsp90
Actin
Actin
124
90
72
54
42
16
124
90
72
54
42
32
27
20200218-3
β-catenin
Actin
0 0.5 1 2.5
0 0.5 1 2.5
HCC-1806
HCC-1806
Cpd 6 (μM), 48h
178
124
90
72
54
42
32
124
90
72
54
42
32
20200428-5

Fig. 5B

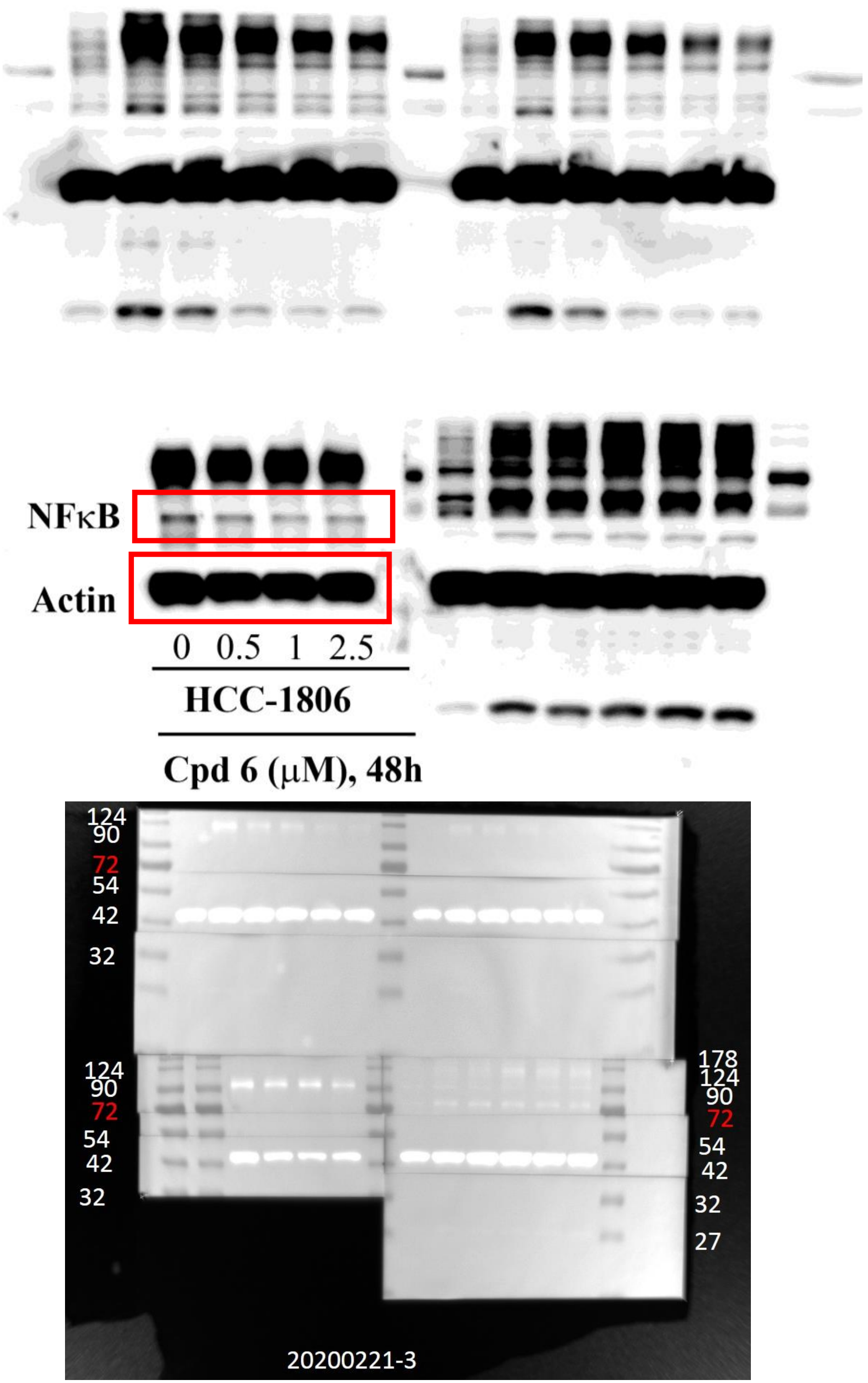

NFκB
Actin
0 0.5 1 2.5
HCC-1806
Cpd 6 (μM), 48h
124
90
72
54
42
32
124
90
72
54
42
32
178
124
90
72
54
42
32
27
20200221-3

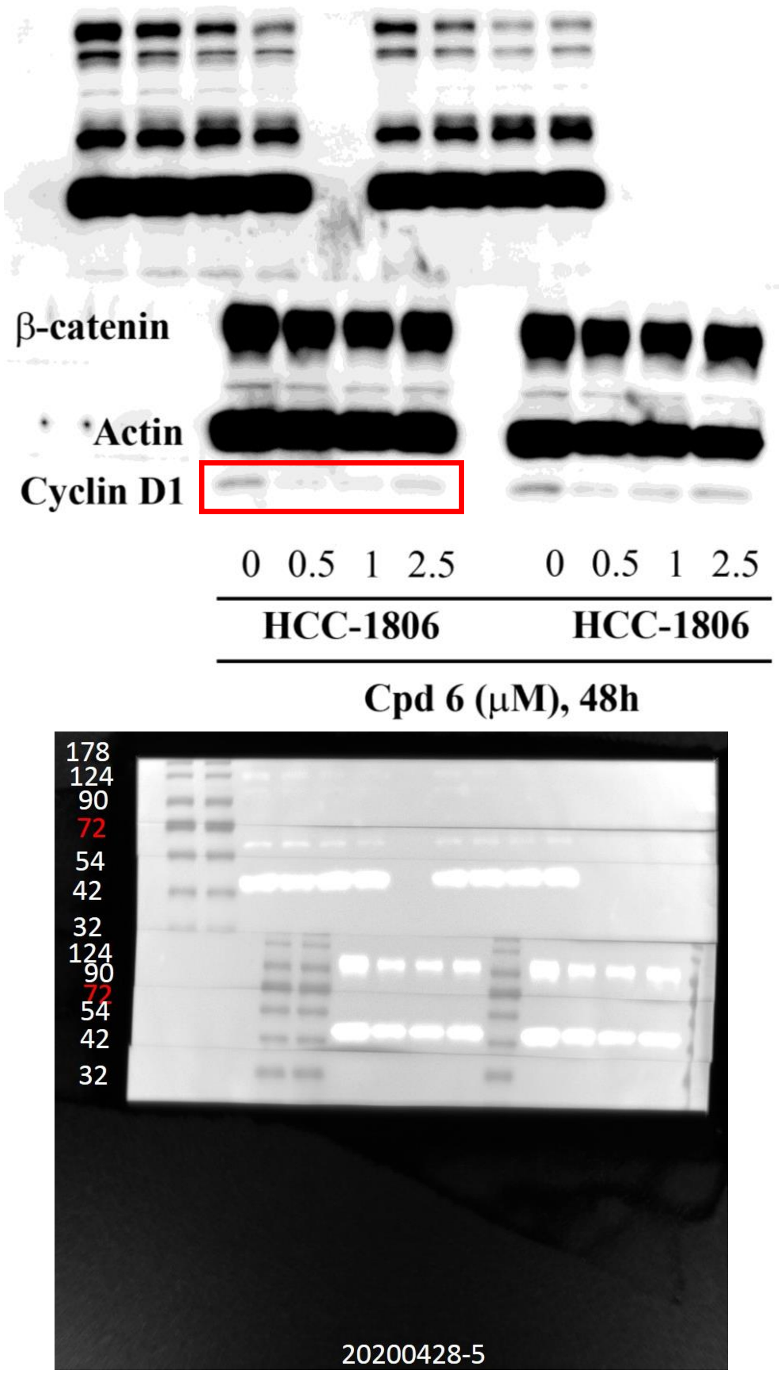

β-catenin
Actin
Cyclin D1
0 0.5 1 2.5
0 0.5 1 2.5
HCC-1806
HCC-1806
Cpd 6 (μM), 48h
178
124
90
72
54
42
32
124
90
72
54
42
32
20200428-5

Fig. 5C (upper)

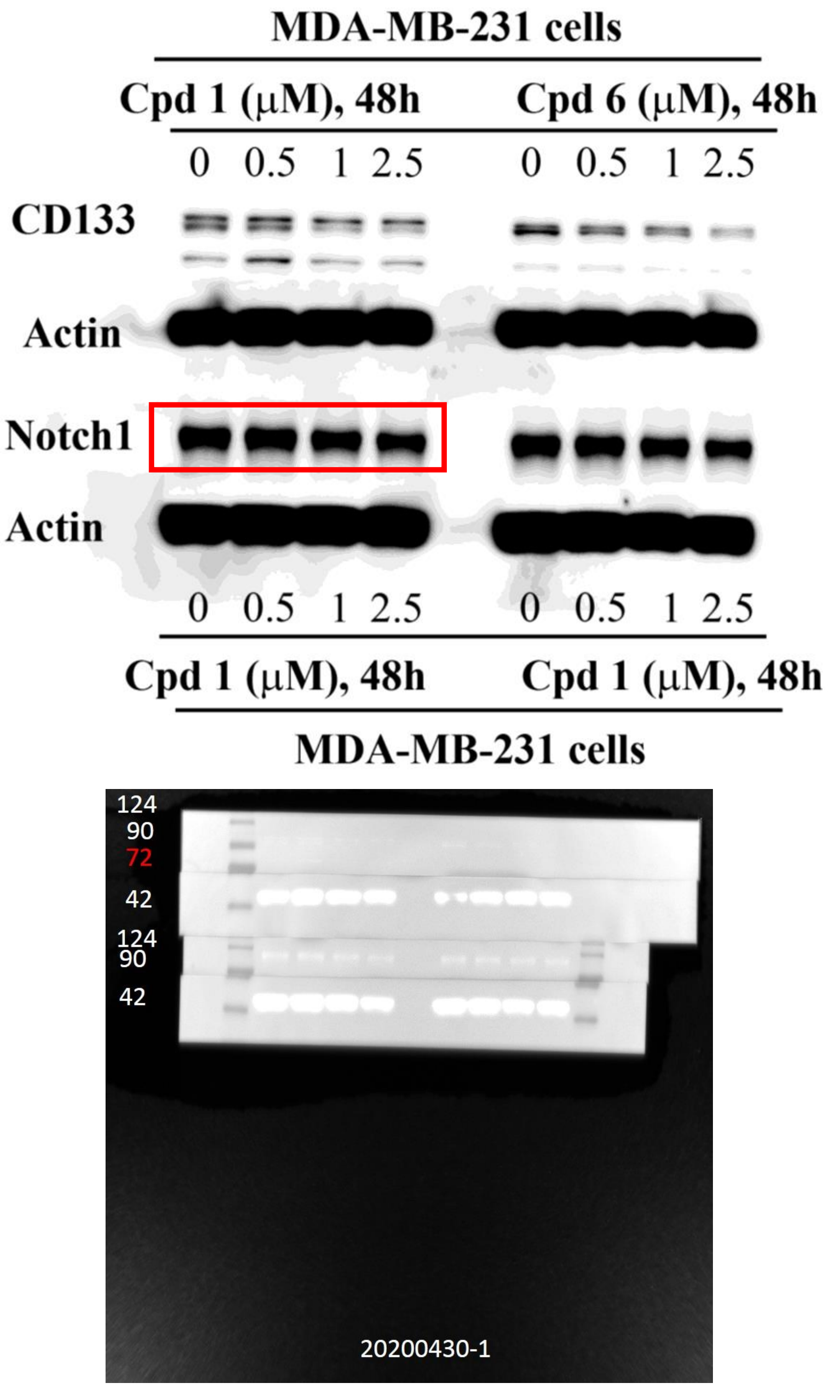
MDA-MB-231 cells
Cpd 1 (μM), 48h
Cpd 6 (μM), 48h
0 0.5 1 2.5
0 0.5 1 2.5
CD133
Actin
Notch1
Actin
0 0.5 1 2.5
0 0.5 1 2.5
Cpd 1 (μM), 48h
Cpd 1 (μM), 48h
MDA-MB-231 cells
124
90
72
42
124
90
42
20200430-1

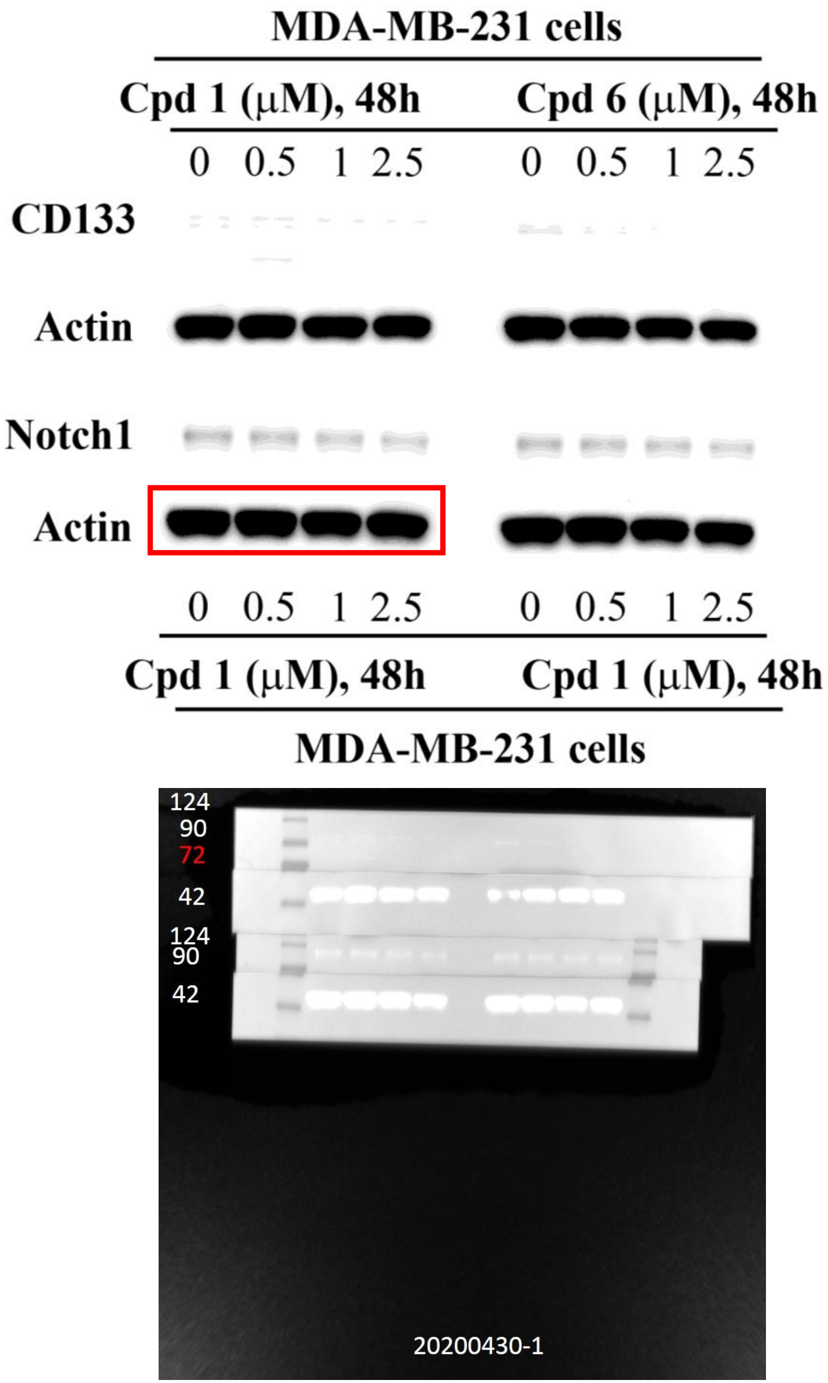
MDA-MB-231 cells
Cpd 1 (μM), 48h
Cpd 6 (μM), 48h
0 0.5 1 2.5
0 0.5 1 2.5
CD133
Actin
Notch1
Actin
0 0.5 1 2.5
0 0.5 1 2.5
Cpd 1 (μM), 48h
Cpd 1 (μM), 48h
MDA-MB-231 cells
124
90
72
42
124
90
42
20200430-1

Fig. 5C (upper)

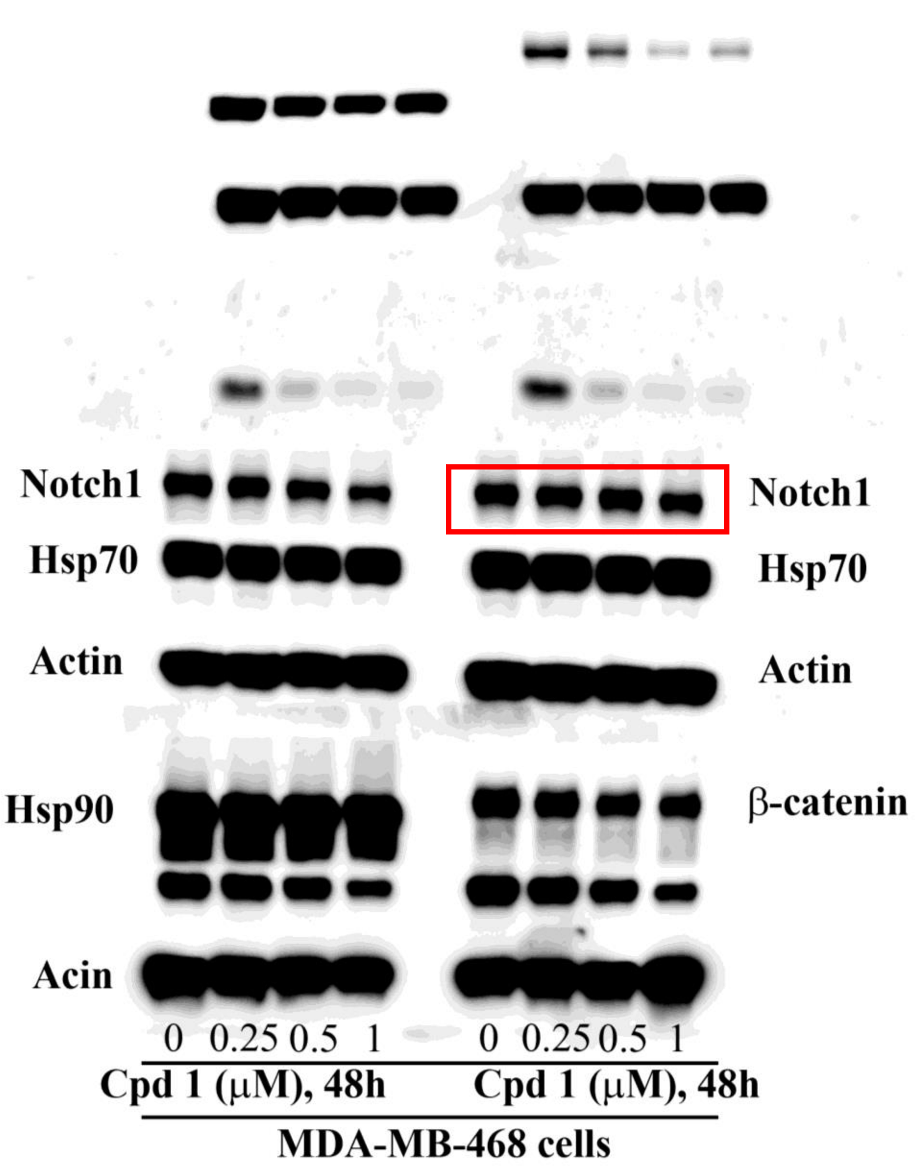

Notch1
Hsp70
Actin
Hsp90
Acin
Notch1
Hsp70
Actin
β-catenin
0 0.25 0.5 1
Cpd 1 (μM), 48h
0 0.25 0.5 1
Cpd 1 (μM), 48h
MDA-MB-468 cells

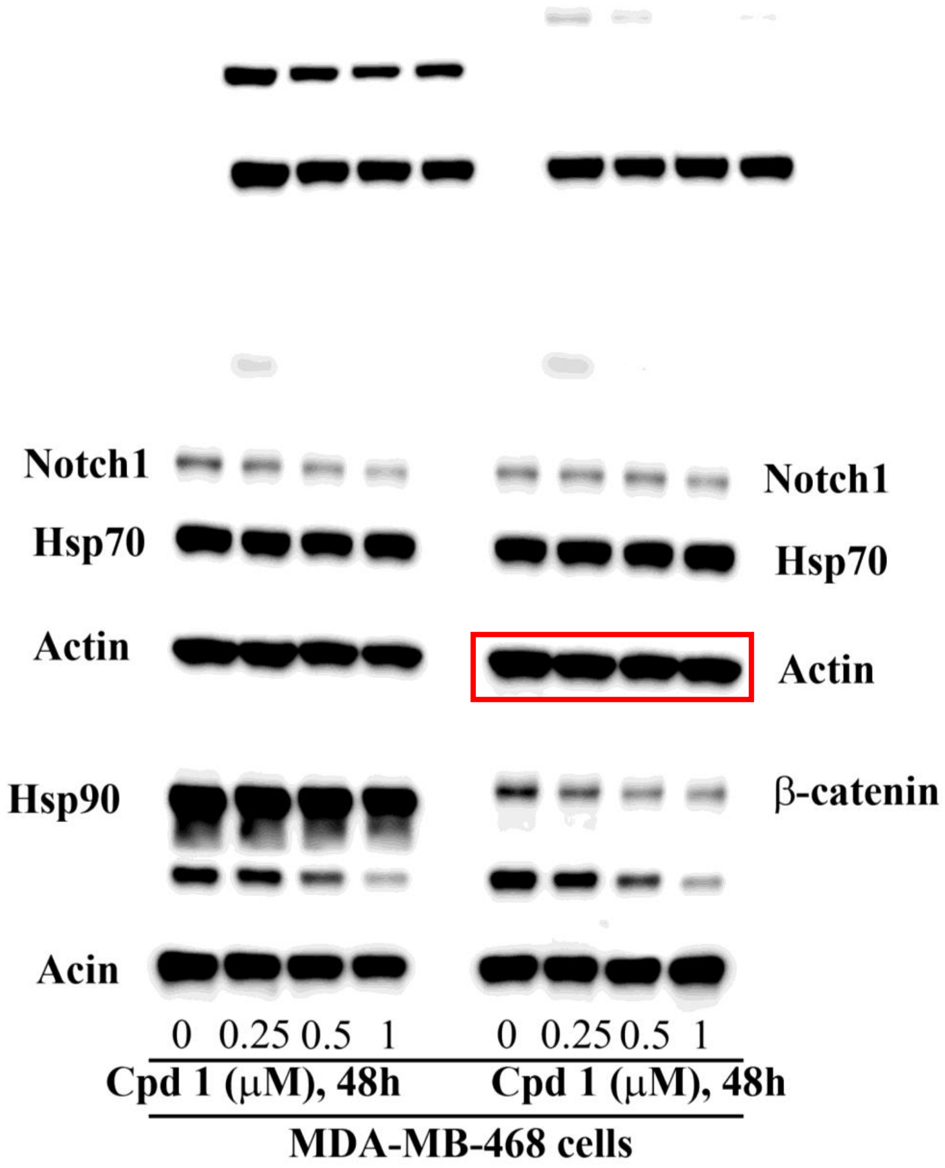

Notch1
Hsp70
Actin
Hsp90
Acin
Notch1
Hsp70
Actin
β-catenin
0 0.25 0.5 1
Cpd 1 (μM), 48h
0 0.25 0.5 1
Cpd 1 (μM), 48h
MDA-MB-468 cells

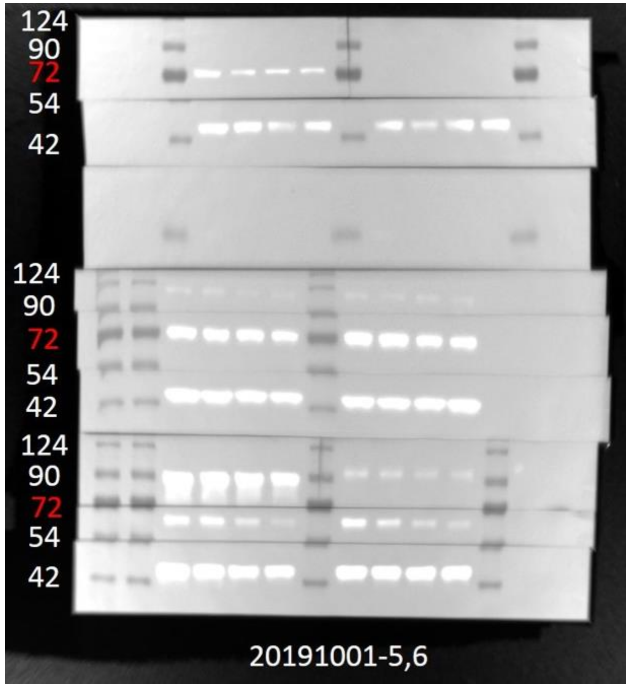

124
90
72
54
42
124
90
72
54
42
124
90
72
54
42
20191001-5,6

Fig. 5C (lower)

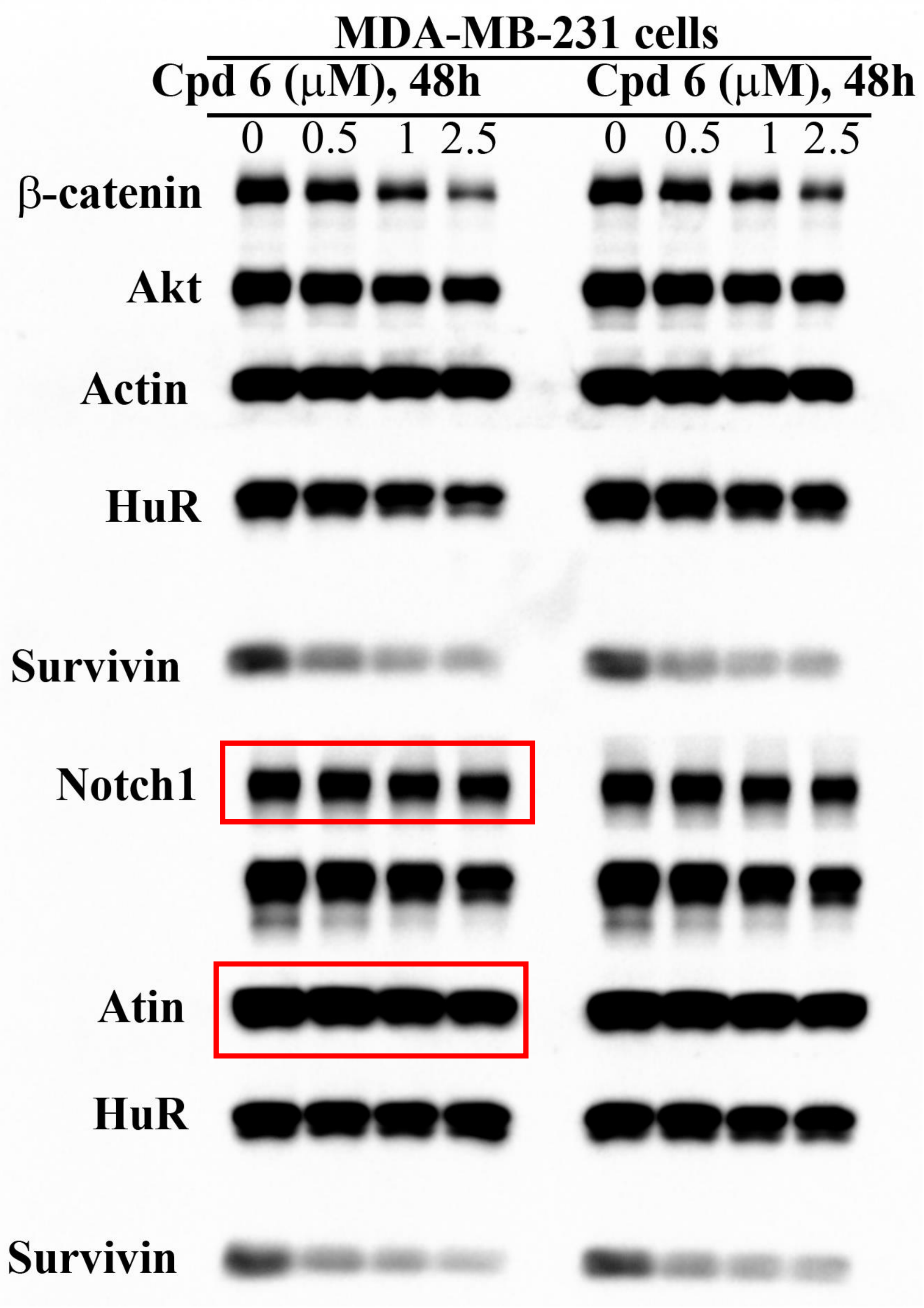
MDA-MB-231 cells
Cpd 6 (μM), 48h
Cpd 6 (μM), 48h
0 0.5 1 2.5
0 0.5 1 2.5
β-catenin
Akt
Actin
HuR
Survivin
Notch1
Atin
HuR
Survivin

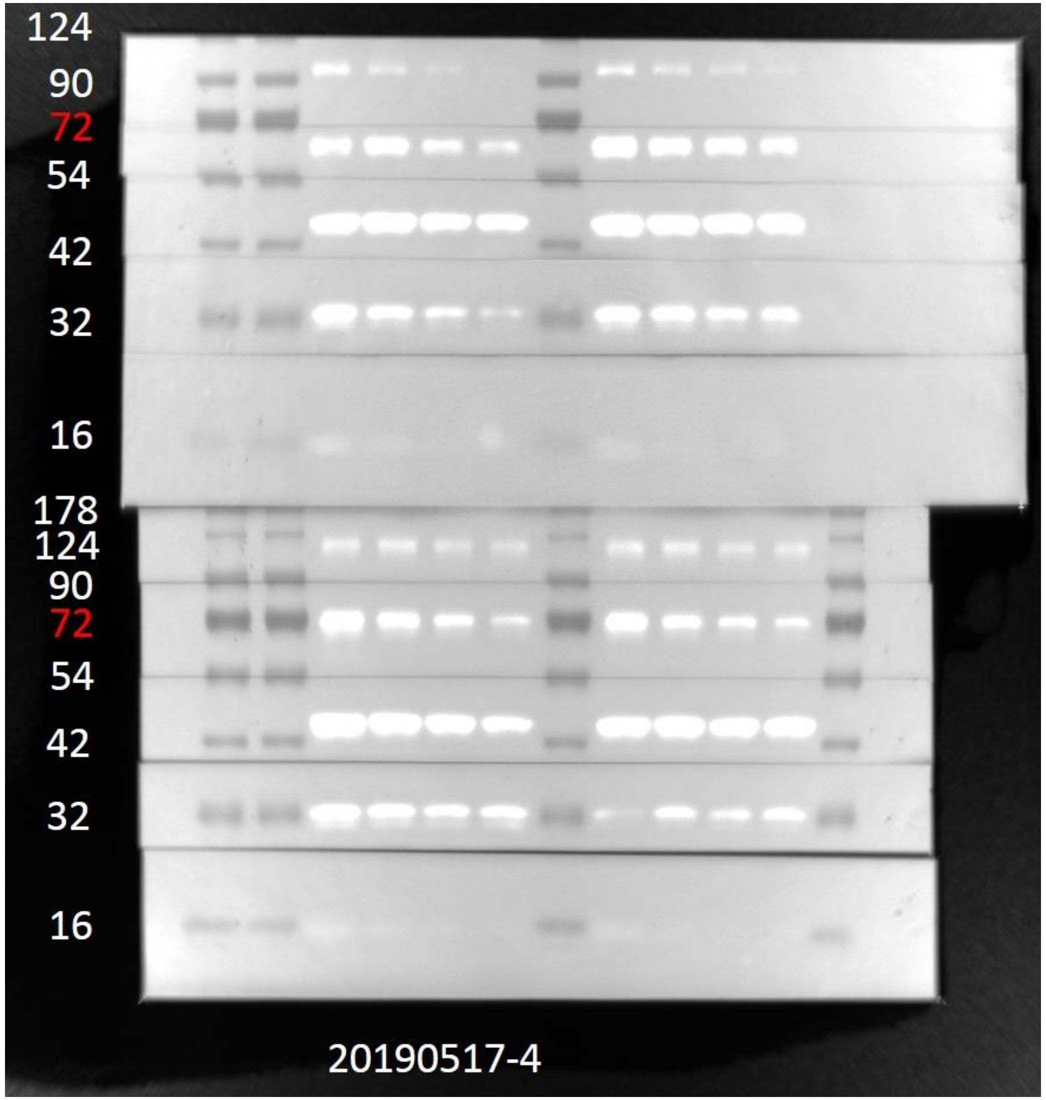
124
90
72
54
42
32
16
178
124
90
72
54
42
32
16
20190517-4

Fig. 5C (lower)

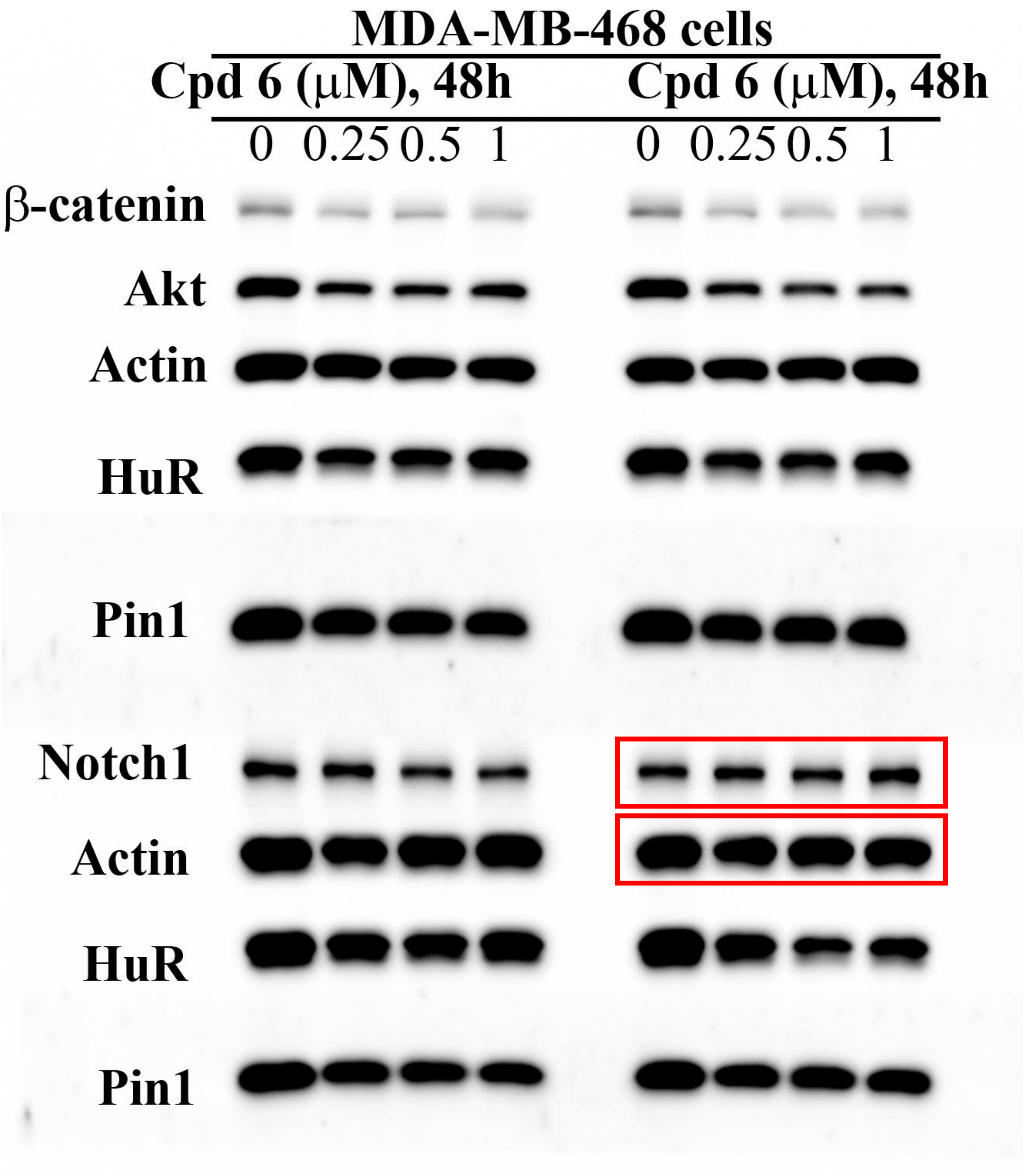
MDA-MB-468 cells
Cpd 6 (μM), 48h
Cpd 6 (μM), 48h
0 0.25 0.5 1
0 0.25 0.5 1
β-catenin
Akt
Actin
HuR
Pin1
Notch1
Actin
HuR
Pin1

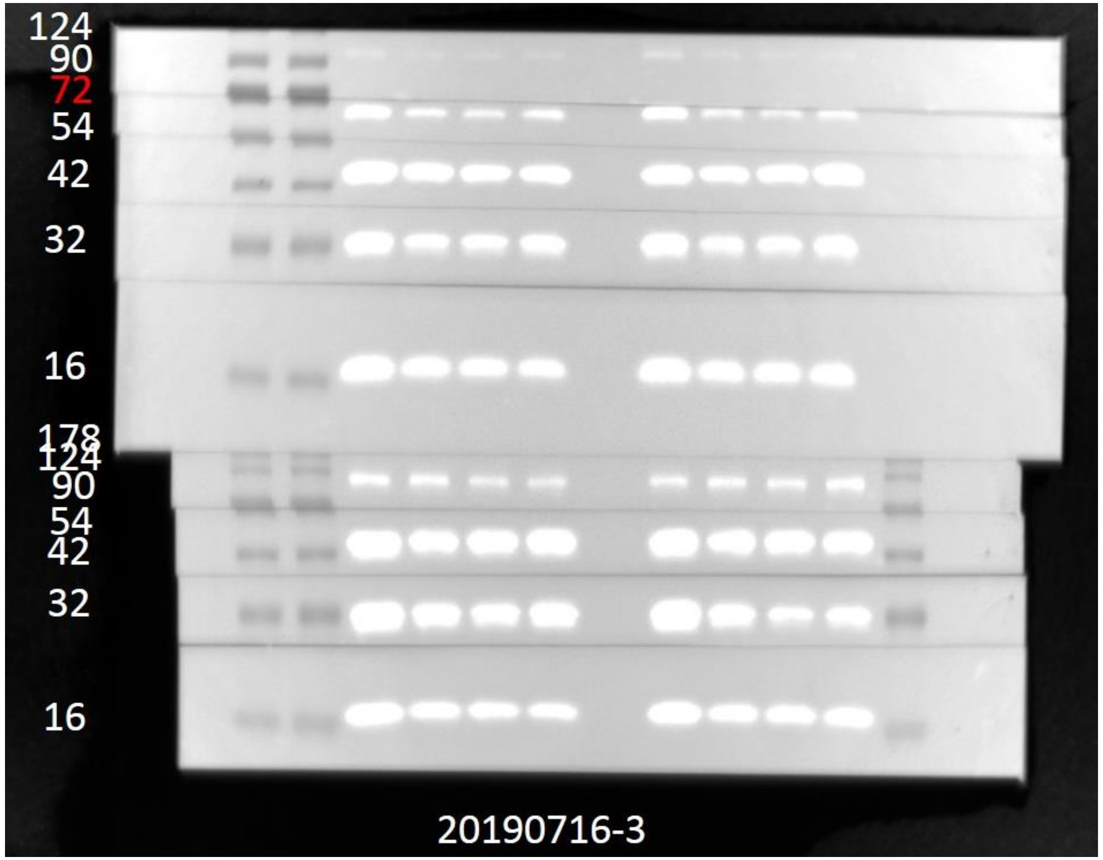
124
90
72
54
42
32
16
178
124
90
54
42
32
16
20190716-3

Fig. 5D

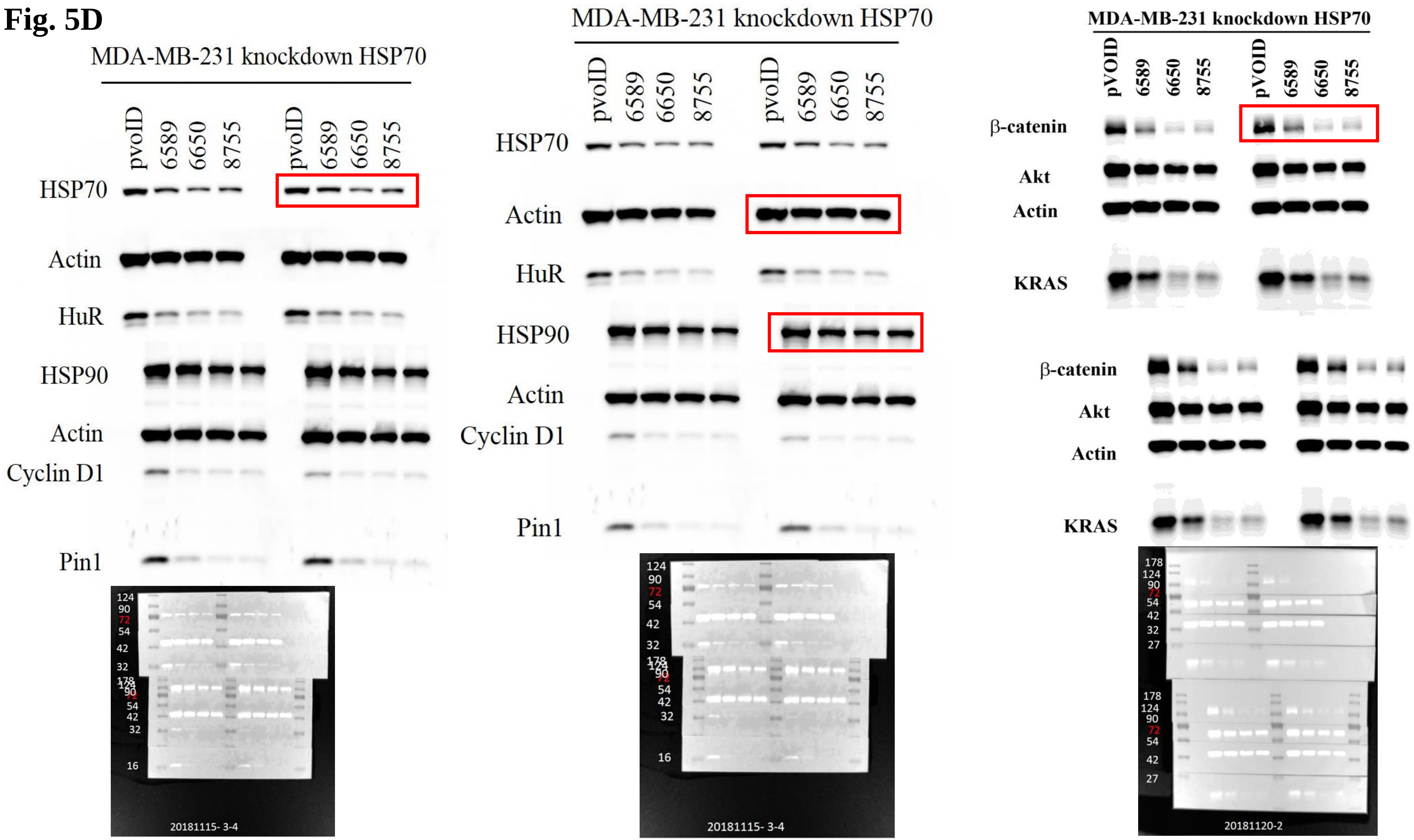
MDA-MB-231 knockdown HSP70
pvoID
6589
6650
8755
pvoID
6589
6650
8755
HSP70
Actin
HuR
HSP90
Actin
Cyclin D1
Pin1
124
90
72
54
42
32
178
124
90
72
54
42
32
16
20181115- 3-4
MDA-MB-231 knockdown HSP70
pvoID
6589
6650
8755
pvoID
6589
6650
8755
HSP70
Actin
HuR
HSP90
Actin
Cyclin D1
Pin1
124
90
72
54
42
32
178
124
90
72
54
42
32
16
20181115- 3-4
MDA-MB-231 knockdown HSP70
pVOID
6589
6650
8755
pVOID
6589
6650
8755
β-catenin
Akt
Actin
KRAS
β-catenin
Akt
Actin
KRAS
178
124
90
72
54
42
32
27
178
124
90
72
54
42
27
20181120-2

Fig. 5D

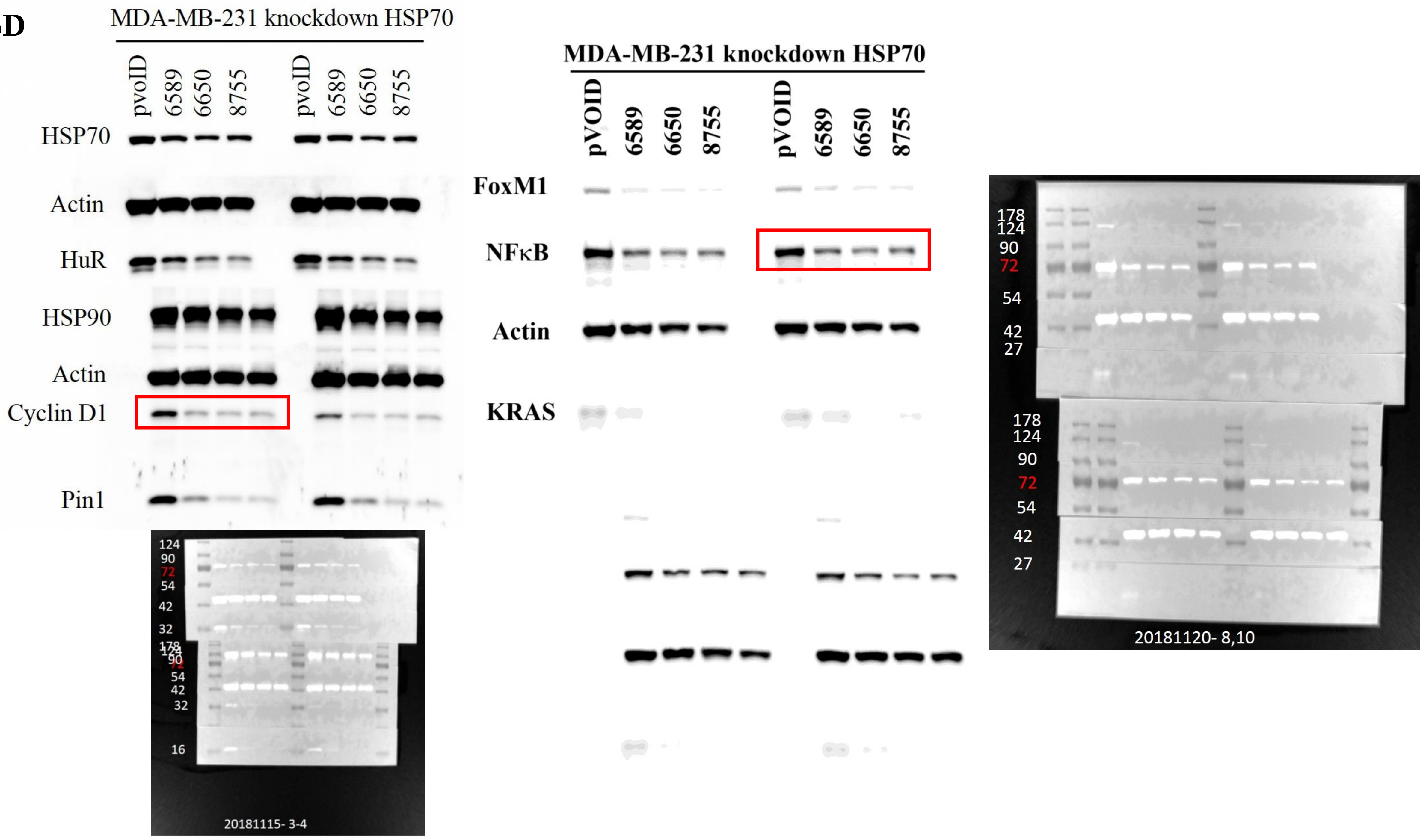
MDA-MB-231 knockdown HSP70
pvoID
6589
6650
8755
pvoID
6589
6650
8755
HSP70
Actin
HuR
HSP90
Actin
Cyclin D1
Pin1
124
90
72
54
42
32
178
124
90
72
54
42
32
16
20181115- 3-4
MDA-MB-231 knockdown HSP70
pVOID
6589
6650
8755
pVOID
6589
6650
8755
FoxM1
NFκB
Actin
KRAS
178
124
90
72
54
42
27
178
124
90
72
54
42
27
20181120- 8,10

Fig. 6A

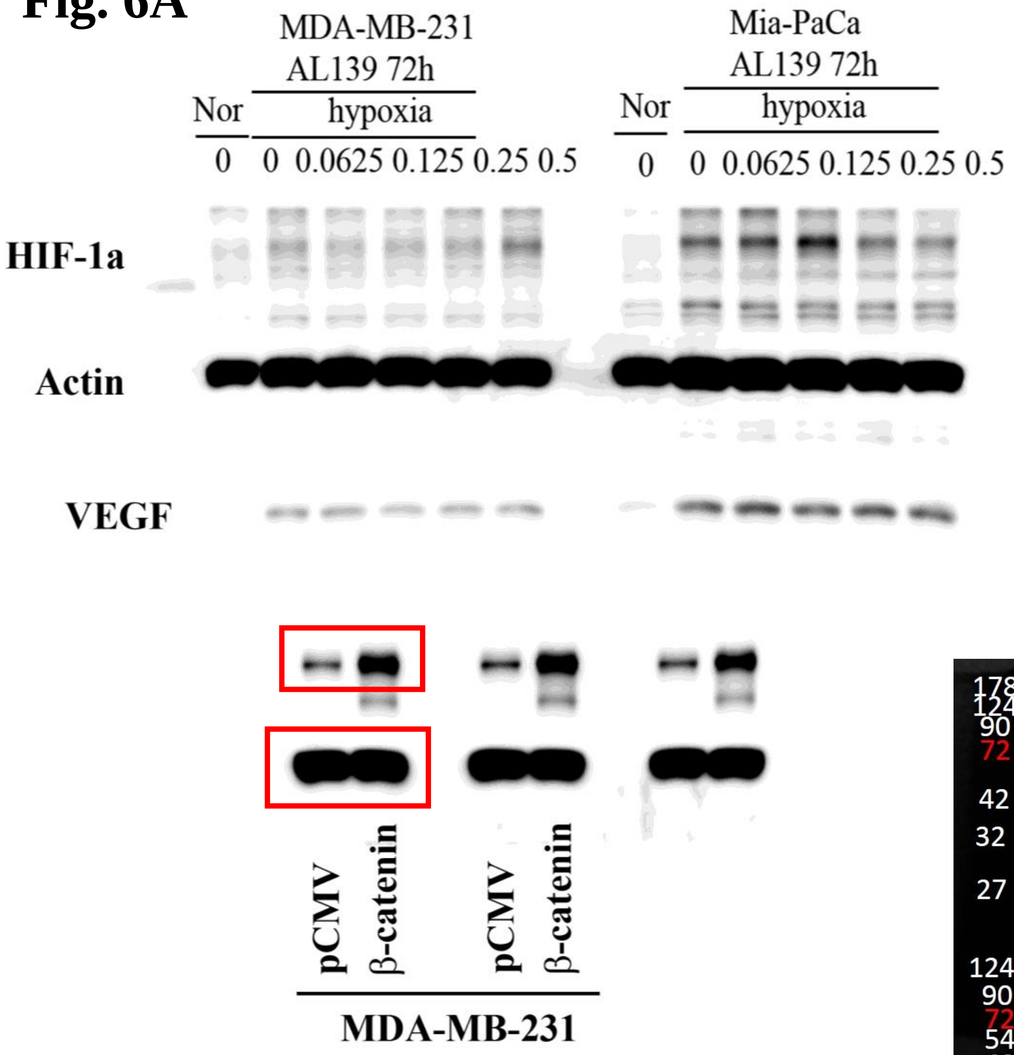

MDA-MB-231
AL139 72h
Nor
hypoxia
0
0 0.0625 0.125 0.25 0.5
Mia-PaCa
AL139 72h
Nor
hypoxia
0
0 0.0625 0.125 0.25 0.5
HIF-1a
Actin
VEGF
pCMV
β-catenin
pCMV
β-catenin
MDA-MB-231

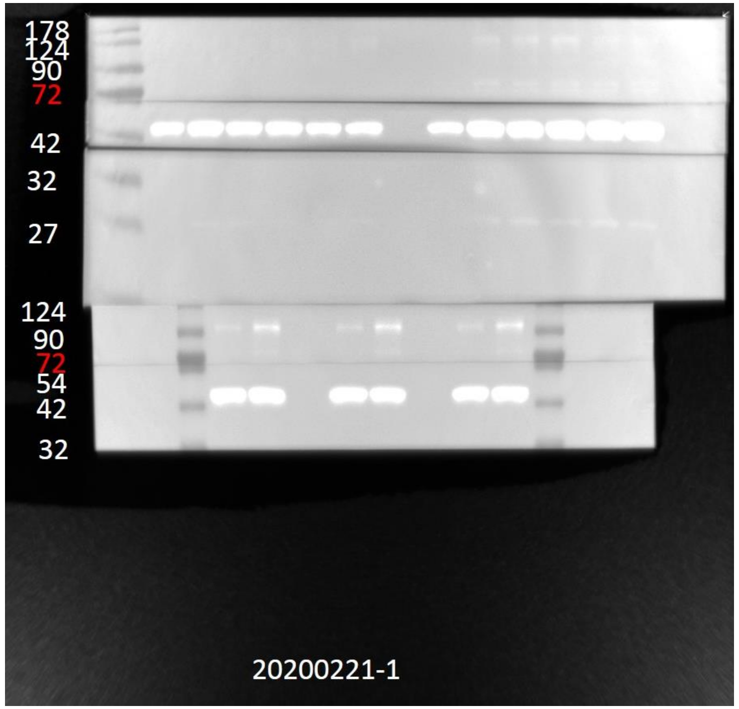

178
124
90
72
42
32
27
124
90
72
54
42
32
20200221-1

Fig. 6B

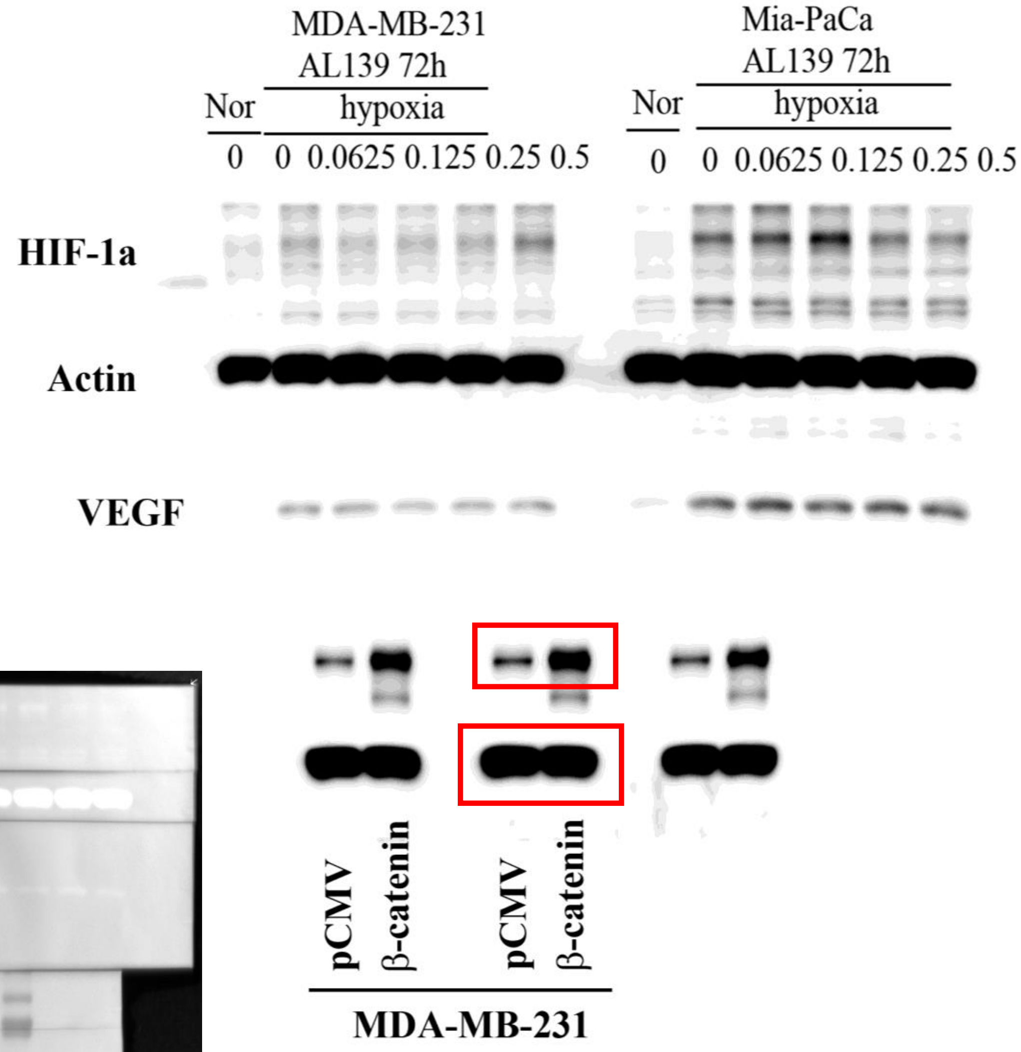

MDA-MB-231
AL139 72h
Nor
hypoxia
0
0 0.0625 0.125 0.25 0.5
Mia-PaCa
AL139 72h
Nor
hypoxia
0
0 0.0625 0.125 0.25 0.5
HIF-1a
Actin
VEGF
pCMV
β-catenin
pCMV
β-catenin
MDA-MB-231
